# Notch-ing up knowledge on molecular mechanisms of skin fibrosis: focus on the multifaceted Notch signalling pathway

**DOI:** 10.1186/s12929-021-00732-8

**Published:** 2021-05-09

**Authors:** Angelo Giuseppe Condorelli, May El Hachem, Giovanna Zambruno, Alexander Nystrom, Eleonora Candi, Daniele Castiglia

**Affiliations:** 1 Genodermatosis Unit, Genetics and Rare Diseases Research Division, Bambino Gesù Children’s Hospital, IRCCS, Piazza Sant’ Onofrio 4, 00165 Rome, Italy; 2 Dermatology Unit and Genodermatosis Unit, Genetics and Rare Diseases Research Division, Bambino Gesù Children’s Hospital, IRCCS, Piazza Sant’ Onofrio 4, 00165 Rome, Italy; 3 Department of Dermatology, Medical Faculty, Medical Center, University of Freiburg, Freiburg, Germany; 4 Department of Experimental Medicine, University of Rome “Tor Vergata”, via Montpellier, 1, 00133 Rome, Italy; 5 IDI-IRCCS, via Monti di Creta 104, 00167 Rome, Italy; 6 Laboratory of Molecular and Cell Biology, IDI-IRCCS, via Monti di Creta 104, 00167 Rome, Italy

**Keywords:** Dermal fibroblasts, Hypertrophic scar, Keloid, Systemic sclerosis, Epidermolysis bullosa, Transforming growth factor-β1, Extracellular matrix, JAG1, NOTCH1, Gamma-secretase inhibitor

## Abstract

Fibrosis can be defined as an excessive and deregulated deposition of extracellular matrix proteins, causing loss of physiological architecture and dysfunction of different tissues and organs. In the skin, fibrosis represents the hallmark of several acquired (e.g. systemic sclerosis and hypertrophic scars) and inherited (i.e. dystrophic epidermolysis bullosa) diseases. A complex series of interactions among a variety of cellular types and a wide range of molecular players drive the fibrogenic process, often in a context-dependent manner. However, the pathogenetic mechanisms leading to skin fibrosis are not completely elucidated. In this scenario, an increasing body of evidence has recently disclosed the involvement of Notch signalling cascade in fibrosis of the skin and other organs. Despite its apparent simplicity, Notch represents one of the most multifaceted, strictly regulated and intricate pathways with still unknown features both in health and disease conditions. Starting from the most recent advances in Notch activation and regulation, this review focuses on the pro-fibrotic function of Notch pathway in fibroproliferative skin disorders describing molecular networks, interplay with other pro-fibrotic molecules and pathways, including the transforming growth factor-β1, and therapeutic strategies under development.

**Supplementary Information:**

The online version contains supplementary material available at 10.1186/s12929-021-00732-8.

## Background

Tissue homeostasis and function ground on the equilibrium between extracellular matrix (ECM) synthesis and degradation. However, in specific biological contexts the remodelling cycle of the ECM transiently shifts towards one of its two phases, a phenomenon that occurs for example during the physiological wound healing process. A wide range of genetic, immunological, metabolic and environmental cues can perturb the homeostatic turnover of ECM, leading to fibrosis: the disproportionate and disorganized accumulation of ECM components, mainly collagens, in various organs [1, 2]. Dermal fibrosis underlies a large and heterogeneous spectrum of pathological conditions which can affect exclusively the skin or where the skin represents one of the targeted organs within the context of a multisystem disorder. Fibrotic diseases involving the skin include disorders due to an aberrant cutaneous wound healing process (e.g. hypertrophic scars and keloids), systemic sclerosis, dystrophic epidermolysis bullosa, chronic graft-versus-host disease, eosinophilic fasciitis and nephrogenic systemic fibrosis [3–5].

Progresses in deciphering the cellular and molecular bases of fibrosis have revealed that a multitude of mechanisms are able to trigger or sustain fibrotic events in a context-dependent fashion. Transforming growth factor (TGF)-β1 represents the best-characterized player in fibrosis onset and maintenance [6, 7]. However, other factors, ranging from an inflammatory *milieu* to additional pro-fibrotic pathways, can overlap or support the TGF-β1 activity shaping the fibrotic processes and determining a wide range of disease manifestations [8–10].

Notch signalling is a highly conserved, ubiquitous, cell–cell communication pathway involved in cell fate, proliferation and tissue homeostasis both in embryonic development and adult life [11]. Notch activation requires binding between the Notch receptor exposed on the surface of a “signal-receiving cell” and the Notch ligand on a juxtaposed “signal-sending cell”. Receptor-ligand interactions commit Notch receptor to a two-step proteolytic cascade generating a transcriptionally active intracellular fragment. Given its pleiotropic actions, the correct timing and magnitude of Notch activation are crucial to avoid detrimental consequences. In particular, gene mutations in the Notch core pathway members and network components are causative of neoplastic disorders [12–14] and of a number of rare, mainly developmental, diseases affecting almost all body districts [11, 15, 16]. In addition, Notch signalling is dysregulated during the onset and course of a variety of diseases, including fibrosis. Notch can exert a pro-fibrotic role in lung, kidney, liver and skin by regulating myofibroblast activation and epithelial-to-mesenchymal transition (EMT), or by dialoguing with other potent fibrogenic pathways, in particular the TGF-β1 signalling [17].

This review focuses on the pro-fibrotic role of Notch pathway in acquired and inherited fibroproliferative disorders affecting the skin. After briefly introducing the general mechanisms of Notch activation and regulation, we will summarize Notch involvement in tissue fibrosis with a specific emphasis on the Notch cross-talk with ECM components and the TGF-β signalling pathway. Thereafter we will discuss Notch role in skin physiopathology, focusing on the most recent findings on the fibrogenic activity of Notch in systemic sclerosis, hypertrophic scars, keloids and dystrophic epidermolysis bullosa. Finally, the current molecular approaches to counteract Notch activation will be reviewed.

## Notch activation, processing and regulation

### Notch signalling cascade: from receptor-ligand binding to transcriptional outcomes

**Fig. 1:**
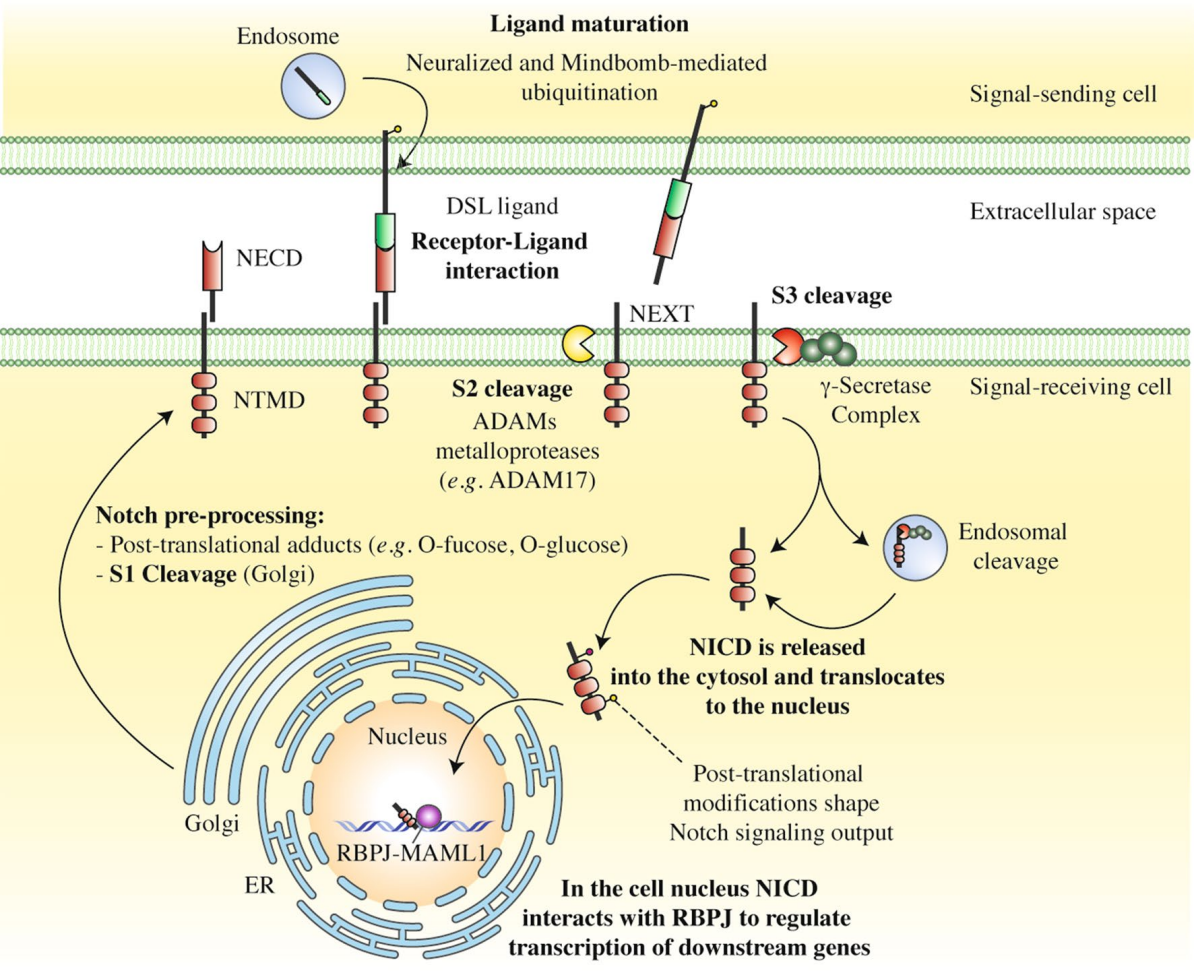
An overview of Notch maturation, activation and processing. Before integration into the plasma membrane, Notch receptor is decorated with different glycans by a complex series of enzymatic reactions occurring within the endoplasmic reticulum (ER) or the Golgi network. Post-translational adducts determine a differential responsiveness of Notch-expressing cells to the ligands. Thereafter, Notch receptor is cleaved at the level of the S1 cleavage site (S1) by a furin-like convertase residing in the trans-Golgi network. The cleavage results in the formation of a heterodimeric receptor, consisting of a Notch extracellular domain (NECD) and a Notch transmembrane domain (NTMD) held together by Ca^2+^-dependent ionic bonds [22, 23]. Similarly, also Notch ligand undergoes a “maturation process” consisting in its endocytosis, ubiquitination by the Neuralized and Mindbomb E3 ubiquitin ligases and “recycling” to the plasma membrane [24]. Notch ligands belong to the Delta/Serrate/LAG2 (DSL) protein family. After ligand binding, the mature Notch receptor is subjected to two successive proteolytic cleavages (S2 and S3 cleavage). The first cleavage is exerted by an ADAM metalloprotease (*e.g.* ADAM17) close the transmembrane domain to generate the Notch extracellular truncation (NEXT) fragment (S2 cleavage). The second is operated by the γ-secretase complex within the transmembrane domain of the NEXT fragment (S3 cleavage) or in endosomes, to dump into the cytoplasm the biologically active Notch intracellular domain (NICD) (reviewed in [11]). In the cell nucleus, NICD forms a trimeric complex with RBPJ and MAML1, which initiates transcription of Notch downstream target genes

In mammals, the Notch family is made of four Notch receptors (Notch 1–4) and five ligands (Jagged 1 and 2, Delta-like 1, 3 and 4) differently expressed depending on the specific cell type and its biological context. Notch activation usually requires the binding between a Notch receptor and a Notch ligand exposed on two different, neighboring cells. Ligand engagement triggers two subsequent proteolytic cleavages (S2 and S3 cleavages) of Notch receptor to release a biologically active Notch intracellular domain (NICD), which translocates into the nucleus where it activates the transcription of its downstream targets, thereby mediating a multitude of biological effects (Fig. 1) [11]. A “pulling force” exerted by the Notch ligand toward its tied receptor drives Notch activation by unmasking an ADAM (short for A Disintegrin And Metalloproteinase) metalloprotease cleavage site at the level of the Notch regulatory region (NRR), close to Notch receptor transmembrane segment [18, 19]. The NRR site is targeted by ADAM proteins, which operate the first ligand-induced proteolytic cleavage (S2 cleavage) and produce the Notch extracellular truncation (NEXT) fragment. In the second activation step, the NEXT fragment is cleaved by the γ-secretase complex (S3 cleavage) to generate the NICD protein product (Fig. 1) [20, 21].

Although the mature Notch receptor usually interacts with its Notch ligand by direct cell–cell contact (trans-activation), several evidences indicate that Notch signalling can also take place over long distances through dynamic filopodia [25, 26], basal protrusions in epithelial cells [27] or exosomes [28, 29].

In the nucleus, NICD interacts with the DNA-binding protein RBPJ (recombination signal binding protein for immunoglobulin kappa J region) and the co-activator Mastermind-like protein 1 (MAML1) creating a ternary complex able to trigger target gene transcription. A growing body of evidence indicates that RBPJ acts as transcription repressor in the absence of NICD, whilst the recruitment of NICD determines the transition from an “OFF” to a transcriptionally active state by complex mechanisms including (i) the displacement of a set of RBPJ-bound co-repressors, (ii) modifications in RBPJ affinity/specificity for selected DNA loci or (iii) chromatin rearrangements favouring accessibility and function of distal enhancer elements [30–32]. In mammals, the prototypical bona fide Notch target genes are represented by the basic helix-loop-helix (bHLH) transcriptional factors of the hairy and enhancer of split (HES) family, including *HES1* and *HES5*, as well as by the *HEY* gene family (HES related with YRPW motif) such as *HEY1* [30]. HES and HEY family members act as transcriptional repressors of gene expression by direct and indirect mechanisms including the recruitment of histone deacetylases or the heterodimeric binding of specific bHLH activators, respectively [33]. HES and HEY factors critically drive differentiation of various cell types, and, for these reasons, are tightly regulated and subjected to rapid cycles of activation and repression [33]. Beyond *HES* and *HEY* genes, multiple factors including (i) the context-dependent accession by Notch transcriptional complex to specific DNA response elements, (ii) the NICD cooperation with different co-activators, and (iii) the effects of post-translational modifications (PTMs) and epigenetic modulators (see below) underlie the qualitatively divergent Notch-derived transcriptomes and determine the exceptionally wide range of Notch-based biological responses [11, 34].

### Regulation of Notch pathway by post-translational modifications

Notch signalling is under the control of multiple mechanisms in each phase of its complex intracellular routes [35]. A variety of regulatory processes modulate Notch receptor and ligand expression, maturation, localization, trafficking and stability prior, during and after the ligand-receptor interaction at the plasma membrane. An important role in controlling Notch pathway is played by post-translational modifications (PTMs) that occur at different stages of Notch member molecular life course. Indeed, both Notch receptors and ligands can undergo glycosylation, methylation, hydroxylation, acetylation, ubiquitination, and, not least, phosphorylation [34]. Notch PTMs cooperate to finely tune the pathway functioning or to terminate the signal [36]. For a recent review of PTM-mediated regulation of Notch pathway see [34]. Among PTMs, ubiquitination of NICD by the ubiquitin E3 ligase FBXW7 (also known as SEL10) has been validated as a key mechanism to switch-off Notch effects [37]. Of note, FBXW7-dependent ubiquitination requires the concomitant phosphorylation of specific amino acid residues within the Pro-Glu-Ser-Thr (PEST) domain of NICD, by different kinases, including the Down-syndrome-associated kinases DYRK1A and DYRK1B [38] and the cyclin-dependent kinase 8 (CDK8) [39]. Thus, PTMs and their interplay represent an additional wall to break down in understanding the complexity of Notch cascade, and potential druggable targets to modulate Notch activity in disease conditions [34].

### Epigenetic regulation of Notch signalling

microRNAs (miRNAs or miRs) are a class of non-coding RNAs (nc-RNAs) that regulate gene expression at the posttranscriptional level. Each miRNA binds to specific messenger RNAs (mRNAs), thus indicated as miRNA targets, leading to their degradation or translational inhibition. miRNAs are involved in almost all physiological and pathological events, including the fine regulation of the Notch pathway. Indeed, an ever-growing number of miRNAs have been reported as modulators of the Notch pathway [40–43]. A list of miRNAs experimentally-validated by gene reporter assay as negative regulators of Notch receptors and ligands is available as Supplementary Information [see Additional file 1: Table S1].

The impaired regulation of miRNAs targeting Notch pathway members contributes to many disease conditions, including fibrosis of various organs [43–47]. Notably, the Notch signalling pathway can also up- or down-regulate the expression of specific miRNAs. For instance, Notch activation in vascular smooth muscle cells (VSMCs) results in a direct, RBPJ-dependent, up-regulation of miR-143/145 cluster which in turn cooperates with Jagged 1 (JAG1)/Notch signalling to promote VSMC contractile phenotype [48]. On the other hand, the Notch-RBPJ-MAML1 complex can repress miRNA expression, as described in CD4 T cells for miR-29 family members [49].

Long nc-RNAs (lncRNAs) are a broad and heterogeneous class of RNA molecules (> 200 nucleotides in length) including intergenic transcripts and sense/antisense RNAs overlapping protein-coding genes. Functional studies revealed that lncRNAs can act both as enhancers and repressors of gene expression through in cis and in trans mechanisms. However, the roles and biological relevance of the vast majority of them remain elusive [50]. As for the interplay between Notch signalling and lncRNA activity, it has been shown that the lncRNA NEAT1 (Nuclear-Enriched Abundant Transcript 1) controls miR-129-5p levels and indirectly regulates the abundance of NOTCH1, a miR-129-5p target, in rat astrocytes [51]. In neural progenitors, the lncRNA LncND (Neuro Development) controls miR-143-3p activity, and in turn the expression levels of NOTCH1 and NOTCH2, two miR-143-3p targets [52]. On the other hand, the Notch pathway drives the expression of the pro-tumorigenic lncRNA LUNAR1 (Leukemia-Associated Non-coding IGF1R Activator RNA 1) in T-ALL [53] and colorectal cancer [54]. Review articles focused on the crosstalk between Notch signalling pathway and lncRNAs have been recently published [55, 56].

Besides miRNA- or lncRNA-based regulatory mechanisms, other processes such as histone [57–60] and mRNA modifications, the latter known as epitranscriptome, have been demonstrated to regulate Notch activity in a cell-specific manner [61, 62]. In particular, the reversible methylation of the N6 position of specific adenosine bases (m^6^A) within target mRNAs and nc-RNAs in eukaryotic cells has recently emerged as a pervasive modulator of gene expression, with roles in health and disease conditions. Briefly, m^6^A influences the mRNA structure and the binding of specific regulatory proteins, with implications in splicing, nuclear retention, mRNA stability and translation efficiency [62–66]. As for the interplay between Notch and m^6^A, a recent study revealed that METTL3 (methyltransferase like 3), a subunit of the N6-methyltransferase complex, is able to methylate several Notch transcripts and potentiate Notch activity in glioma stem-like cells [62].

## Notch pathway in tissue fibrosis

Fibrosis is a pathological condition marked by excessive deposition of fibrous connective tissue in an injured or inflamed tissue. It can affect all organs resulting in disruption of the physiological tissue architecture and function [67]. A wound healing (WH) response ensues any tissue injury to rapidly restore homeostasis. The WH process is classically resumed in four successive but overlapping phases: haemostasis, inflammation, new tissue formation (or proliferative phase) and remodelling. In the WH context, fibrosis is due to several interlinked mechanisms that affect the proper magnitude and spatiotemporal sequence of the WH phases, leading to a chronic WH response [68, 69], with continued tissue damage, repair and regeneration. Different acute or chronic stimuli including infections, autoimmune and inflammatory reactions and mechanical injury contribute to fibrosis onset, often in a disease-dependent manner [67].

The phenoconversion of fibroblasts (FBs) and other mesenchymal precursor cells into a highly specialized cell type called myofibroblast is a crucial process that triggers and sustains fibrogenesis in all fibrotic diseases. Myofibroblasts are characterized by contractile and secretory abilities given by the production of specific contractile proteins (*e.g.* α-SMA, α-smooth muscle actin) and secretion of ECM matricellular proteins [70, 71]. Of note, many of the classical fibrogenic signalling pathways such as TGF-β, platelet-derived growth factor (PDGF), WNT and hedgehog (Hh) are strictly connected and cooperate to induce myofibroblast differentiation and persistence, driving disease progression and maintaining it over time [2, 7, 8].

### Notch and fibrosis

Notch signalling is emerging as a potent inducer of fibrosis in liver, lung, kidney and skin [17, 72–74]. The Notch cascade is involved in FB proliferation [75], myofibroblast differentiation [74, 76], contractile phenotype induction and acts synergistically with other pro-fibrotic pathways, primarily the TGF-β signalling. These pro-fibrotic features are particularly evident in VSMCs, where: (i) the JAG1-NOTCH1-RBPJ axis and the myocardin (MYOCD) signalling synergistically activate expression of smooth muscle cell (SMC) marker genes such as myosin, heavy polypeptide 11, smooth muscle (MYH11) and transgelin (TAGLN) [77] and (ii) JAG1/NOTCH3 interactions lead to the induction of contractile marker proteins (α-SMA and calponin—CNN1) and, in turn, of the contractile phenotype in in vitro SMCs-endothelial cells 3D coculture models [78] as well as in VSMC disorders driven by inactivating mutations in the NOTCH3/TGF-β regulator gene *HtrA* serine peptidase 1 (HTRA1) [79]. Moreover, Noseda and coll. demonstrated that the human *ACTA2* gene, encoding α-SMA, contains a RBPJ consensus binding site, whose activation is necessary and sufficient to obtain a Notch-mediated α-SMA transcription in endothelial cells and primary FBs [80–82]. In addition to its direct effects in regulating myofibroblast differentiation as well as the synthetic/proliferative and contractile features of activated FBs, Notch signalling possesses an important role in inflammation and EMT [83–85], two processes involved in fibrosis outcome. However, studies in this field, in relation to fibrotic mechanisms, are still limited.

### Notch cross-talk with the extracellular matrix and its pro-fibrotic components

The extracellular matrix (ECM) is the non-cellular constituent of all tissues and organs. It contains a hydrated multifaceted mixture of macromolecules (*e.g.* fibrous glycoproteins, glycosaminoglycans, proteoglycans) which is synthetized by all resident cells, especially by FBs [86–88], in a tissue- and context-dependent manner [89, 90]. ECM works as a structural scaffold but also importantly as a biologically active system, able to modulate the behaviour of the surrounding cells and orchestrate a plethora of processes including cell proliferation, motility, differentiation, polarity and WH. Given its pleiotropic roles, ECM homeostasis is crucial for health maintenance [91–93]. Indeed, ECM architecture and composition are altered in multiple pathological conditions, including fibrosis [94–96]. Specific to fibrosis, it is well established that ECM composition and mechanical properties strongly impact on the bio-availability and activity of key anti- and pro-fibrotic factors involved in fibrosis, first and foremost the fibrogenic growth factor TGF-β1 [94, 95, 97–99].

At the cellular level, surface receptors and proteins decorating the membranes act as effective “mechanosensors” able to intercept biochemical and biophysical ECM modifications (*e.g.* composition, force and rigidity—the so-called ECM stiffness) and to convert them into molecular and functional inputs through the induction of intracellular signalling cascades [100].

The pro-fibrotic Notch pathway is also emerging as a “sensing system” able to intracellularly transmit a variety of microenvironmental cues, including chemical and physical ECM modifications [101]. On the other hand, ECM modulates Notch signalling activation through direct and indirect mechanisms involving both core ECM components and ECM-related pathways. A pertinent example is represented by MAGP-2 (Microfibril Associated Glycoprotein-2)—a microfibril/elastin network structural component also involved in fibrosis [102]—which directly interacts with the tandem EGF-like repeats in DSL (Delta/Serrate/LAG2) ligands and NOTCH1 receptor. MAGP-2-NOTCH1 interactions promote the receptor heterodimer dissociation and its activation through an ADAM-independent mechanism [103, 104]. On the contrary, in endothelial cells MAGP-2 co-operates with the integrin αVβ3 to recruit the c-SRC kinase which phosphorylates N1ICD at specific tyrosine residues, leading to a reduction of its half-life and transcriptional ability [105, 106]. Additional ECM proteins able to modulate Notch activation via direct or indirect routes include periostin (POSTN), an ECM matricellular protein which binds NOTCH1 and preserves its expression in stress conditions [107], type I and type IV collagens [108] and laminin-111 [109].

### Notch and TGF-β1: the fibrogenic dialogue

The TGF-β pathway is a paradigm of signal transduction mechanisms initiated by the activation of a serine/threonine kinase receptor at the plasma membrane. Briefly, TGF-β cascade is triggered by the binding between a member of the TGF-β and BMP (Bone Morphogenetic Protein) ligand subfamilies, and a ligand-specific heteromeric receptor complex [110]. Then, the signal is propagated by the receptor-mediated recruitment and phosphorylation activation of the cytoplasmic SMAD proteins (SMADs) (TGF-β canonical pathway) or by alternative molecular cascades, in particular the MAPK (Mitogen Activated Protein Kinase) signalling (TGF-β non-canonical pathway) [110–112]. Phosphorylated SMAD2 and SMAD3 proteins form a complex with SMAD4 to convey signals from TGF-β receptors into the nucleus, where SMADs act as transcriptional factors. The intricate interplay of ligand and receptor types, SMADs and cofactors as well as of signal-driven transcription factors (SDTFs) and lineage-determining transcription factors (LDTFs) underlies the various TGF-β-dependent transcriptional outcomes in relation to specific cell contexts and biological conditions [110].

Notably, TGF-β1 signalling plays an outstanding role in fibrosis of several tissues and organs, due to the potent induction of ECM protein synthesis and myofibroblast differentiation [113, 114]. Importantly, TGF-β-mediated modifications of the ECM composition and properties (e.g. ECM stiffness) contribute to perpetuate, in a vicious-cycle, myofibroblast activity and TGF-β production [97, 114].

Several reports highlighted a cooperation between different Notch and TGF-β pathway members in a variety of cellular contexts and biological processes [83, 115–119], including fibrosis.

The Notch pathway members JAG1 and HES1, are early transcribed in response to TGF-β stimulation in HaCaT keratinocytes [115]. Similarly, the TGF-β/Notch axis synergistically acts to promote the transcription of Hes1 in chicken embryos, neural stem cells, myoblasts and epithelial cells through SMAD3 and SMAD4 activation [83, 120]. Interestingly, SMAD3 is able to directly interact with NICD and can be recruited to RBPJ-binding sites on DNA in the presence of RBPJ and NICD in C2C12 myoblast cells [120]. In the context of fibrosis, the Notch/TGF-β axis cooperates to induce fibrosis in several tissue and organs, and its pharmacological targeting represents a powerful strategy to counteract the fibrogenic process [121–124]. The dialogue between Notch and TGF-β induces the expression of contractile and pro-fibrotic markers (e.g. α-SMA, CNN1 and TAGLN) in different cell types including SMCs [125], mesenchymal stem cells (MSCs) [126], lung FBs [122] and RLE-6TN rat alveolar epithelial cells [127]. In RLE-6TN cells, the cis-elements CArG box (CC(A/T)_6_GG box) and TCE (TGF-β control element) lying in the promoter of *ACTA2* gene are critical for the transcriptional induction of α-SMA in response to both TGF-β and NICD [127]. The well-established role of Notch in contractility-associated gene expression, coupled with its mechanosensitive properties [27] and its TGF-β-mediated induction in response to mechanical stress [128], suggest that the fibrosis-driven activation of Notch signalling could prime or enforce, in a self-powered loop, the fibrotic behaviour of myofibroblasts.

The Hippo member Yes-associated protein 1 (YAP1) is emerging as an important mechanosensitive system, able to convert external ECM-dependent inputs into pro-fibrotic outputs at the intracellular level. In TGF-β1–treated FBs as well as in primary myofibroblasts from patients with Dupuytren disease, a fibroproliferative disorder of the hands and fingers, YAP1 activation leads to induction of a pro-fibrotic phenotype with increased ECM production and cell contractility [129]. Interestingly, the two highly related transcriptional cofactors YAP and TAZ can also interact with the Notch cascade by different modalities in relation to the different cellular contexts. In general, YAP/TAZ can (i) mediate transcriptional regulation of Notch family members; and (ii) co-operate with Notch to transcribe common, downstream target genes [130].

Beyond the TGF-β, a cross-talk between Notch and the fibrogenic Wnt pathway [131] has been shown to occur at multiple levels ranging from a reciprocal regulation to an opposite or synergistic activity [119, 132–137]. Despite the well-established relationship between Notch and Wnt signalling, mechanistic studies exploring their interplay in fibrotic disease models are still missing [138].

## Notch expression and role in skin physiology and pathology

### Notch in keratinocyte differentiation and proliferation

**Fig. 2:**
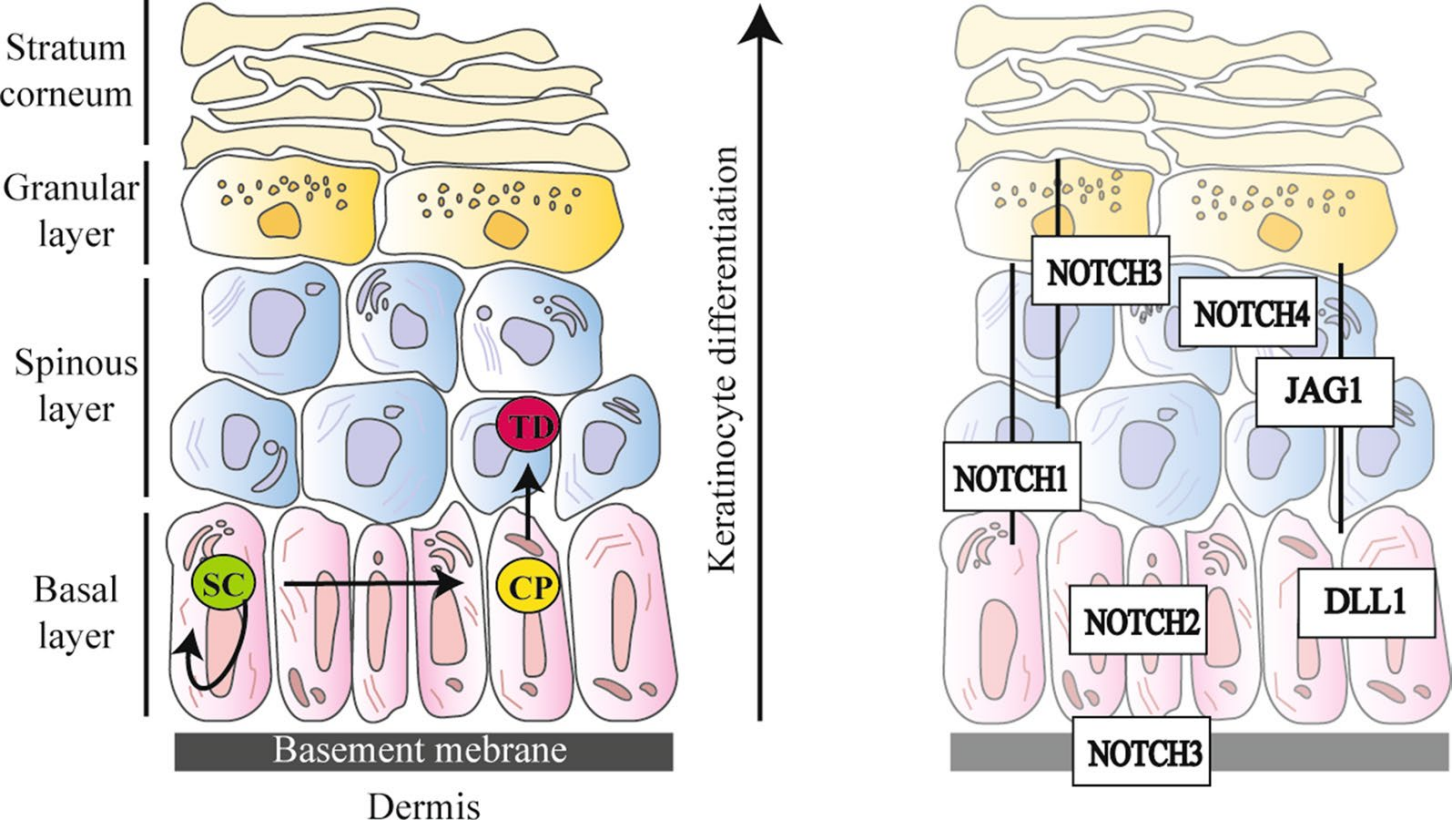
Skin architecture and Notch pathway member distribution. (Left panel) Organization of human skin. The epidermis is the thinnest and most superficial layer of the skin, it is connected to the underlying dermis through the cutaneous basement membrane zone (BMZ), the highly specialized structure which connects the epidermis to the dermis ensuring skin integrity and stability against mechanical insults. Epidermis is arranged into four distinct layers: basal, spinous, granular and stratum corneum (or horny layer). The deepest layer of the epidermis, overlaying the BMZ, is the basal layer which is followed by the spinous and the granular layers whilst the stratum corneum is the outermost. Each layer is typified by keratinocytes (KCs) at various stages of differentiation. KCs of the basal layer are characterized by their ability to proliferate. Indeed, in the basal layer, epidermal stem cells (SCs) divide to self-renew and produce transient amplifying cells (also known as committed progenitors—CPs), which possess a more limited proliferation capability. In the early phases of differentiation, CPs detach from the BMZ and move toward the stratum corneum becoming terminally differentiated cells (TDs) devoid of nuclei. (Right panel) Expression pattern of Notch receptors (NOTCH1-4) and Notch ligands (JAG1 and DLL1) in human skin [143, 144]

From a simplified perspective, human skin is composed of a stratified squamous epithelium, the epidermis, standing above a connective tissue, the dermis (Fig. 2, left panel). Epidermal tissue is continually self-renewing, and keratinocytes (KCs), the major cell type of the epidermis, represent its foremost “shaping force”. Epidermis is organized into four distinct layers typified by KCs at various stages of differentiation (Fig. 2, left panel). In brief, moving upward from the basal layer to the top of the epidermis (i.e. the horny layer) KCs undergo a complex series of molecular events that progressively modify their features, such as morphology and keratin type expression and organization, and commit the cell to terminal differentiation (TD) (Fig. 2, left panel). Epidermal homeostasis rests on a perfect equilibrium between KC proliferation and differentiation programs. The Notch signalling pathway exerts a crucial role in regulating and maintaining skin homeostasis, orchestrating KCs differentiation at the level of inter-follicular epidermis (IFE) and hair follicles (HFs), and finally working in epithelial barrier formation [139–142]. The analysis of Notch signalling components in skin revealed that Notch receptors and ligands exhibit stringent qualitative and quantitative expression patterns within the different areas of IFE and HFs [140, 143, 144] (Fig. 2, right panel). As for the specific effects of Notch receptors and ligands in human KCs, functional investigations revealed that NOTCH1 reduces the proliferative rate of stem cells (SCs) compartment, while NOTCH2 and NOTCH3 cooperate to induce TD [145]. The anti-proliferative role of NOTCH1 has been also documented in mouse epidermis [146] and is in line with its tumour suppressor function in squamous cell carcinoma (SCC) [147]. For these reasons, dysregulation of Notch signalling can contribute to the pathomechanisms of various skin disorders and syndromes marked by an altered KC proliferation/differentiation rate including Adams-Oliver syndrome—a congenital disorder characterized by terminal transverse limb malformations, skin and skull bone defects—as well as psoriasis and atopic dermatitis [148].

### Notch in fibroproliferative skin diseases

**Fig. 3:**
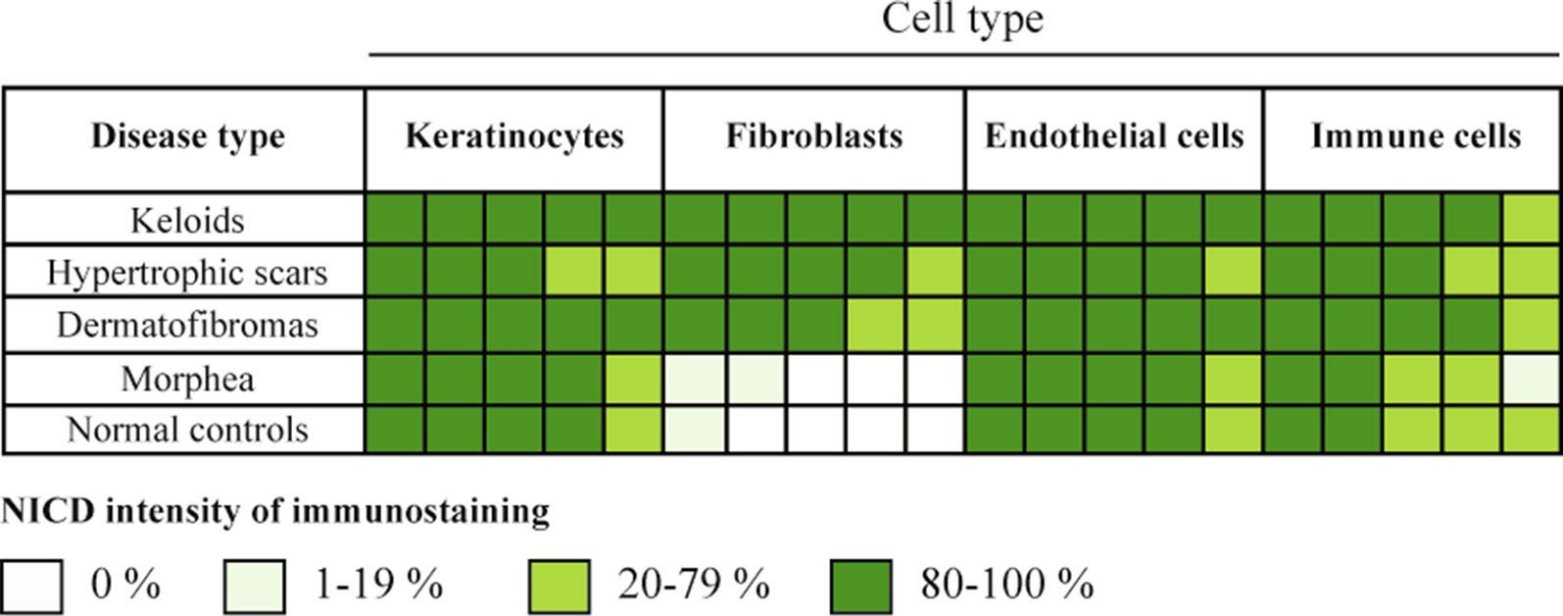
NOTCH1 activation in fibroproliferative skin diseases. Immunohistochemical staining pattern showing the expression levels of the cleaved, biologically active NOTCH1 protein (NICD, Notch intracellular domain) in skin biopsies from patients affected with four different fibroproliferative diseases and normal controls. In general, NICD is constitutively expressed in keratinocytes, endothelial cells and immune cells of patients affected with the various fibroproliferative disorders and healthy biopsies, with no significant variation among them. As for NICD expression in fibroblasts, it was generally low in morphea and barely detected in healthy controls. On the other hand, Notch pathway is activated in fibroblasts from keloids, hypertrophic scars and dermatofibromas. Figure adapted from [149]

Multiple lines of evidence revealed that Notch activation is involved in the pathogenesis of skin fibroproliferative diseases, both acquired (e.g. dermatofibromas, hypertrophic scars, keloids and systemic sclerosis) and inherited (i.e. dystrophic epidermolysis bullosa, DEB). A comparative immunohistochemical analysis of skin biopsies from patients affected with different fibroproliferative diseases revealed that NICD staining, suggestive of Notch pathway activation, varies in a cell-dependent manner according to the disease types (Fig. 3) [149]. In particular, abundant expression of NICD was present in FBs from keloids, hypertrophic scars and dermatofibromas, while it was barely detectable in FBs from normal control skin. The following paragraphs describe the most recent advances in Notch-mediated mechanisms of fibrosis in skin diseases.

#### Systemic sclerosis

Systemic sclerosis (SSc) is an autoimmune connective tissue disorder of unknown etiology affecting the skin, muscles and multiple internal organs, characterized by a highly heterogeneous evolution and outcomes. In SSc patients, the hyperactivated immune response against a multitude of autoantigens leads to vascular dysfunction [150], inflammation and fibrosis of the skin and visceral organs [151]. The molecular mechanisms underlying SSc onset are intricate, and many pathways contribute to its pathogenesis, including the signalling cascade initiated by TGF-β [152] and the Notch pathway [153].

In vivo and in vitro evidences showed that Notch signalling is activated in SSc and drives fibrosis [76, 154]. High levels of NICD and JAG1 have been found in lesional skin biopsies as well as in cultured skin FBs from SSc patients. Interestingly, in SSc fibrotic skin the expression of JAG1 ligand is particularly prominent in inflammatory infiltrates, suggesting that T-cells might contribute to activate Notch cascade in resident FBs [76]. Pharmacological and siRNA-mediated inhibition of Notch signalling in primary SSc FBs reduces *COL1A1* and *COL1A2* expression and α-SMA abundance. On the other hand, healthy FBs treated with the recombinant JAG1 protein (Jag1-Fc) show an increased release of ECM and fibroblast-to-myofibroblast differentiation [76]. Inflammation- and ROS-depended hyperactivation of ADAM17 could represent a mechanism underlying the aberrant levels of NICD in SSc skin [154–156].

As for epigenetic involvement in SSc fibrosis, Wasson and coll. recently described the up-regulation of the lncRNA HOX transcript antisense RNA (HOTAIR) in primary SSc FBs, and validated its involvement in fibrogenesis via the EZH2-mediated activation of Notch signalling [47]. In addition, exosomes (EXOs)—small membrane-bound vesicles of endocytic origin involved in cell–cell communication [157, 158]—derived from serum [159] and neutrophils of SSc patients [160] display a specific set of differentially expressed miRNAs and lncRNAs involved in fibrosis and regulation of pro-fibrotic pathways, including Notch [159, 160]. Imatinib is a tyrosine kinase (TK) inhibitor, with an anti-inflammatory and anti-fibrotic role in preclinical and clinical models of SSc and other disorders [161, 162]. Notably, recent pharmacokinetic analyses revealed that the Notch pathway also controls imatinib uptake in SSc FBs through the reduction of expression levels of key organic molecule transporters, such as the multidrug and toxin extrusion transporter MATE1 [163]. Finally, specific *NOTCH3* polymorphisms correlate with an increased susceptibility to develop various forms of SSc, suggesting that they can prime disease pathomechanisms or modulate clinical manifestations of fibrosis [164].

#### Hypertrophic scar

Hypertrophic scar (HS), clinically appearing as a raised scar confined to the site of injury, is a common WH complication, deriving from aberrant abnormal proliferative and remodelling phases on a background of genetic susceptibility [9, 165]. HS is typified by dermal alterations, in particular FB hyperproliferation, overproduction of ECM and by the persistence of α-SMA-positive myofibroblasts [166]. In addition, the epidermis is thickened and its major constituents, the KCs, are activated and undergo accelerated differentiation [166, 167]. Finally, the KC-FB cross-talk plays an important role in HS development [168, 169]. A study by Li and coll. revealed that Notch signalling is activated in the epidermis of HS patients, regulates production of fibrotic factors in KCs, both in vitro and in vivo models, and significantly contributes to scar hyperplasia [168]. Inflammatory burden is one the most important factors in pathological scarring and macrophages are well-known determinants in the pathological drift of the healing process in several skin diseases, including the HS [170]. A recent study showed that blocking Notch activity in macrophages alleviates scar formation by lessening inflammatory response and collagen accumulation [171]. At the same time, in vivo experiments revealed that RBPJ knock-out (KO) mice are characterized by a reduced expression of type I and type III collagens and of several fibrotic markers in the healed skin [171].

Adipose-derived mesenchymal stem cells (AMSCs) are considered promising tools to counteract fibroproliferative disorders such as HS and keloids in accordance to their ability to reduce the production of pro-fibrotic factors and lessen myofibroblast features. Recently, Han and coll. demonstrated that conditioned medium from AMSCs alleviates the fibrotic phenotype (*i.e.* fibrosis-associated ECM synthesis, proliferation, migration) of HS and KD FBs by reducing the activity of TGF-β1 and NOTCH1 cascade [172].

#### Keloid disease

Keloid disease (KD), which appears clinically as a raised scar that grows beyond the injury site and has no tendency to regress, is a benign although disabling fibroproliferative skin disorder derived by an excessive and abnormal WH process [173]. The dermis of KD is characterized by abundant thick hyalinized collagen bundles, also known as “keloidal collagen”, and by the persistence of myofibroblasts, and is surmounted by a thickened epidermis which undergoes accelerated differentiation [166]. Though Notch signalling has been less investigated in KD than in other fibrotic skin diseases, available experimental evidences support its involvement in KD pathomechanisms [174, 175]. A study by Syed and Bayat revealed that (i) NOTCH1, NOTCH2 and JAG1 mRNA and protein levels are significantly up-regulated in KD skin biopsies and primary FBs from KD patients (KD FBs) with respect to healthy tissues and FBs, respectively; (ii) Notch pathway stimulates cell proliferation, migration, invasion and angiogenetic properties of cultured KD FBs; and (iii) Notch activation positively correlates with inflammatory degree in KD tissues, suggesting that immune cells might turn on Notch cascade in vivo [174]. Furthermore, a recent study reported that KD FBs from subjects affected with active KD (*i.e.* patients with a recent keloid and complaining keloid pruritus and pain) exhibit a more prominent NOTCH1 activation as compared to KD patients with stable lesions [175]. In addition, KD FBs were hallmarked by: (i) a reduced autophagic flux, which has been associated with a reduced autophagy-mediated degradation of Notch, and (ii) a NOTCH1-mediated induction of α-SMA, TGF-β3 and NLRP3 (NACHT, LRR and PYD domains-containing protein 3) inflammasome, which primes the inflammatory cascade in KD. Finally, treatment of KD FBs with rapamycin—an inducer of autophagy—determined NOTCH1, and in turn NLRP3, down-regulation in KD FBs [175]. Deregulation of the Notch pathway in KD has been recently described by an integrative analysis of mRNA and miRNA expression levels at the wound site of KD-prone individuals [176].

#### Dystrophic epidermolysis bullosa

Epidermolysis bullosa (EB) embraces a heterogeneous group of inherited skin fragility disorders typified by skin blistering and superficial wounds [177]. The underlying genetic defects are the major determinants of disease extent and clinical phenotype. However, genetic, epigenetic and environmental factors can deviate the expected genotype–phenotype correlations, contributing to disease severity variability in EB individuals carrying the same mutations [178]. The recessive dystrophic EB (RDEB) subtype is caused by biallelic mutations in the *COL7A1* gene, encoding type VII collagen (COL7), the major component of anchoring fibrils which ensure adhesion of the cutaneous basement membrane zone to the dermis. In RDEB patients, loss of the structural function of COL7 disrupts skin resilience to mechanical stress and impairs the WH process. In RDEB, wound sites are enriched in immune cells, bacteria and myofibroblasts that fuel, in a self-renewing loop, the inflammatory burden and the development of inflammation- and injury-driven soft tissue fibrosis [5]. Fibrosis is a regular and devastating disease complication in RDEB patients that leads to joint contractures, hand and foot digit fusion and mitten deformities and favours the onset of aggressive and metastasizing squamous cell carcinomas (SCCs) [179, 180]. Understanding the molecular mechanisms regulating fibrosis in RDEB represents a critical step towards the development of novel therapeutic strategies to counteract disease progression and improve patients’ quality of life. Despite the pro-fibrotic role of Notch signalling in a wide range of fibrotic disorders and the paradigm of RDEB as a powerful model to investigate common mechanisms of fibrosis [5], the involvement of this pathway in EB-associated fibrosis remains almost unexplored.

In a recent study, our group showed for the first time that JAG1 protein levels and the cleaved/activated form of NOTCH1 are increased in RDEB FBs as compared to primary skin FBs from healthy subjects and positively correlate with the abundance of the pro-fibrotic miR-145-5p [45]. In accordance with these findings, our previous genome-wide expression analysis performed on RDEB FBs from a monozygotic twin pair with markedly different phenotypic disease manifestations revealed the up-regulation of JAG1 and Notch family members in the more severely affected twin [178]. We are currently exploring the Notch role in RDEB-associated fibrosis, including its interplay with the TGF-β pathway.

In addition to fibrosis, Notch could be an important factor in regulating RDEB-associated inflammation due its well-established involvement in immune cells development and function [84]. Finally, inactivating mutations in NOTCH1, NOTCH2 and NOTCH4 are recognized genetic determinants in RDEB-associated SCCs [180, 181]. Although the pro-tumorigenic properties of Notch pathway, alone or in cooperation with other signalling mechanisms, are well investigated in human cutaneous malignant melanoma and SCC [146, 182–184], mechanistic studies in RDEB-SCC models are missing.

## Therapeutic routes

The fibrosis-limiting effects of Notch signalling inhibition have been extensively and successfully described in numerous preclinical models. However, safety and therapeutic potential of Notch inhibitors remain to be fully elucidated in the clinical practice, in particular in the context of long-term treatments for chronic diseases such as fibrosis [17, 185]. Of note, the Notch cascade represents a relevant therapeutic target in cancer, where over 70 clinical trials have been registered [186–188]. Interestingly Nirogacestat (PF-03084014), a Notch GSI (γ-secretase inhibitor), entered a phase III clinical study (ClinicalTrials.gov Identifier: NCT03785964) for the treatment of desmoid tumour/aggressive fibromatosis, rare, slow-growing malignancies arising from FBs and characterized by a heterogeneous outcome.

GSIs are a wide family of molecules able to halt γ-secretase enzymes activity [189]. Initially, they were developed to block presenilin 1 and 2, the enzymes responsible for amyloid precursor protein formation in Alzheimer’s disease, but nowadays GSIs represent the prototypical drugs to counteract Notch pathway activation in blood malignancies and solid cancers [188, 190–192]. Unfortunately, GSIs are nonspecific Notch inhibitors and the definition of their therapeutic window is critical to avoid toxic side effects [193]. Indeed, low-dose, combinatorial therapies against the main morphogenic pathways (i.e. Hedgehog, Wnt and Notch) have been shown effective in murine models of SSc, and could represent a safer approach to counteract skin fibrosis in human patients [194].

**Fig. 4:**
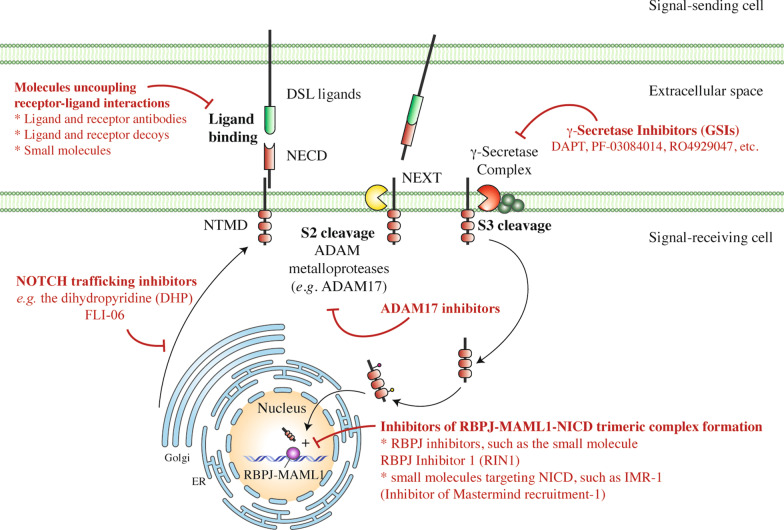
The therapeutic toolkit to modulate Notch signalling. The various strategies to modulate Notch signalling embrace distinct categories of molecules: (i) inhibitors of Notch pre-processing, (ii) molecules uncoupling receptor-ligand interactions including receptor and ligand antibodies and decoys as well as chemical compounds; (iii) inhibitors of NICD formation at the level of the S2 and S3 cleavages, mainly represented by the class of γ-secretase inhibitors (GSIs) and (iv) inhibitors of the trimeric transcriptional complex (RBPJ-MAML1-NICD) assembly. *DSL ligands* Delta/Serrate/LAG2 ligand, *NECD* Notch extracellular domain, *NEXT* Notch extracellular truncation fragment, *NTMD* Notch transmembrane domain, *NICD* Notch intracellular domain

In addition to the GSIs, several molecules able to inhibit the initial steps of Notch trafficking and processing, uncouple receptor-ligand interactions or selectively prevent the interaction between NICDs and their nuclear co-activators have been developed [195–200] (Fig. 4 and Additional file 1: Table S2) and clinical testing is underway for some of them. Non-GSIs Notch inhibitors range from monoclonal antibodies (mAbs) to Notch ectodomain-based molecular decoys. In the clinical practice, antibody- and ectodomain-based inhibition strategies could halt the Notch signalling cascade in a selective manner, avoiding the potential harmful effects given by pan-Notch inhibitors. Moreover, both repurposing drugs such as artesunate, an anti-malarial agent, as well as plant-derived natural products, such as astragaloside, have been reported to down-regulate TGF-β and Notch signalling cascades and lessen pulmonary fibrosis in vitro and in rat models [122, 201].

In conclusion, the intricate mechanisms underlying Notch maturation, processing, regulation and activity represent as many steps of complexity in pathway understanding, but at the same time they make Notch a powerful druggable target in multiple phases of its molecular life, by an ever-growing range of potential therapeutic tools (Fig. 4 and Additional file 1: Table S2).

## Conclusions

Despite Notch signalling represents a timeless research topic in a multitude of physiological and pathological conditions, its mechanism of action remains intricate. Notch-based biological outputs are complex, subjected to a tight regulation and extremely diversified, often in a cell- and context-dependent fashion. This intrinsic complexity makes Notch investigation challenging but fascinating. In the skin, Notch is a well-established regulator of keratinocyte differentiation and, in turn, loss-of-function mutations in genes encoding Notch members are important players in the onset of SCC. On the contrary, though several in vivo and in vitro studies established a role for Notch cascade activation in different fibroproliferative diseases, also in combination with several pro-fibrotic pathways, in particular the TGF-β1, its involvement in FB behaviour appears as yet incompletely investigated. Similarly, interactions between Notch and ECM members in fibroproliferative skin disorders have been described but not fully elucidated. The findings summarized in this review show that the Notch pathway pervasively regulates different aspects of skin homeostasis and its dysregulation can underlie the pathomechanisms of fibrosis. Thus, targeting the Notch cascade could represent a relevant tool for future therapeutic approaches in fibrotic skin disorders.

## Supplementary Information

**Additional file 1: Table S1.** Luciferase reporter assay-validated miRNAs regulating Notch receptors and ligands. **Table S2.** Drugs and compounds targeting Notch pathway.

## Data Availability

Not applicable.

## Abbreviations

α-SMA: Alpha-smooth muscle actin
BMZ: Basement membrane zone
CNN1: Calponin
DAPT: N-[N-(3,5-difluorophenacetyl)-L-alanyl]-S-phenylglycine t-butyl ester
EB: Epidermolysis bullosa
ECM: Extracellular matrix
FBs: Fibroblasts
HS: Hypertrophic scar
KD: Keloid disease
KCs: Keratinocytes
GSI: γ-Secretase inhibitor
lncRNAs: Long non-coding RNAs
MAPKs: Mitogen-activated protein kinases
miRNA (or miR): MicroRNA
NECD: Notch extracellular domain
NEXT: Notch extracellular truncation fragment
NICD: Notch intracellular domain
nc-RNAs: Non-coding RNAs
PTMs: Post-translational modifications
RBPJ: Recombination signal binding protein for immunoglobulin kappa J region (also known as CSL or CBF1)
RDEB: Recessive dystrophic epidermolysis bullosa
SCC: Squamous cell carcinoma
SSc: Systemic sclerosis
TAGLN: Transgelin
TD: Terminal differentiation
TGF-β1: Transforming growth factor-β1
VSMCs: Vascular smooth muscle cells
WH: Wound healing

## References

[1] Do NN, Eming SA (2016). Skin fibrosis: models and mechanisms. Curr Res Transl Med.

[2] Distler JHW, Gyorfi AH, Ramanujam M, Whitfield ML, Konigshoff M, Lafyatis R (2019). Shared and distinct mechanisms of fibrosis. Nat Rev Rheumatol.

[3] Uitto J, Jimenez S (1990). Fibrotic skin diseases. Clinical presentations, etiologic considerations, and treatment options. Arch Dermatol..

[4] Canady J, Karrer S, Fleck M, Bosserhoff AK (2013). Fibrosing connective tissue disorders of the skin: molecular similarities and distinctions. J Dermatol Sci.

[5] Nystrom A, Bruckner-Tuderman L (2018). Injury- and inflammation-driven skin fibrosis: the paradigm of epidermolysis bullosa. Matrix Biol.

[6] Meng XM, Nikolic-Paterson DJ, Lan HY (2016). TGF-beta: the master regulator of fibrosis. Nat Rev Nephrol.

[7] Gyorfi AH, Matei AE, Distler JHW (2018). Targeting TGF-beta signaling for the treatment of fibrosis. Matrix Biol.

[8] Mehal WZ, Iredale J, Friedman SL (2011). Scraping fibrosis: expressway to the core of fibrosis. Nat Med.

[9] Ogawa R (2017). Keloid and hypertrophic scars are the result of chronic inflammation in the reticular dermis. Int J Mol Sci..

[10] Jeljeli M, Riccio LGC, Doridot L, Chene C, Nicco C, Chouzenoux S, Deletang Q, Allanore Y, Kavian N, Batteux F (2019). Trained immunity modulates inflammation-induced fibrosis. Nat Commun.

[11] Siebel C, Lendahl U (2017). Notch signaling in development, tissue homeostasis, and disease. Physiol Rev.

[12] Ellisen LW, Bird J, West DC, Soreng AL, Reynolds TC, Smith SD, Sklar J (1991). TAN-1, the human homolog of the Drosophila notch gene, is broken by chromosomal translocations in T lymphoblastic neoplasms. Cell.

[13] Ferrando AA (2009). The role of NOTCH1 signaling in T-ALL. Hematol Am Soc Hematol Educ Program..

[14] Ranganathan P, Weaver KL, Capobianco AJ (2011). Notch signalling in solid tumours: a little bit of everything but not all the time. Nat Rev Cancer.

[15] Masek J, Andersson ER (2017). The developmental biology of genetic Notch disorders. Development.

[16] Mitchell E, Gilbert M, Loomes KM (2018). Alagille syndrome. Clin Liver Dis.

[17] Hu B, Phan SH (2016). Notch in fibrosis and as a target of anti-fibrotic therapy. Pharmacol Res.

[18] Meloty-Kapella L, Shergill B, Kuon J, Botvinick E, Weinmaster G (2012). Notch ligand endocytosis generates mechanical pulling force dependent on dynamin, epsins, and actin. Dev Cell.

[19] Gordon WR, Zimmerman B, He L, Miles LJ, Huang J, Tiyanont K, McArthur DG, Aster JC, Perrimon N, Loparo JJ, Blacklow SC (2015). Mechanical allostery: evidence for a force requirement in the proteolytic activation of Notch. Dev Cell.

[20] Schroeter EH, Kisslinger JA, Kopan R (1998). Notch-1 signalling requires ligand-induced proteolytic release of intracellular domain. Nature.

[21] Mumm JS, Schroeter EH, Saxena MT, Griesemer A, Tian X, Pan DJ, Ray WJ, Kopan R (2000). A ligand-induced extracellular cleavage regulates gamma-secretase-like proteolytic activation of Notch1. Mol Cell.

[22] Rand MD, Grimm LM, Artavanis-Tsakonas S, Patriub V, Blacklow SC, Sklar J, Aster JC (2000). Calcium depletion dissociates and activates heterodimeric notch receptors. Mol Cell Biol.

[23] Yugawa T, Nishino K, Ohno S, Nakahara T, Fujita M, Goshima N, Umezawa A, Kiyono T (2013). Noncanonical NOTCH signaling limits self-renewal of human epithelial and induced pluripotent stem cells through ROCK activation. Mol Cell Biol.

[24] Weinmaster G, Fischer JA (2011). Notch ligand ubiquitylation: what is it good for?. Dev Cell.

[25] Cohen M, Georgiou M, Stevenson NL, Miodownik M, Baum B (2010). Dynamic filopodia transmit intermittent Delta-Notch signaling to drive pattern refinement during lateral inhibition. Dev Cell.

[26] Cheng D, Yan X, Qiu G, Zhang J, Wang H, Feng T, Tian Y, Xu H, Wang M, He W, Wu P, Widelitz RB, Chuong CM, Yue Z (2018). Contraction of basal filopodia controls periodic feather branching via Notch and FGF signaling. Nat Commun.

[27] Hunter GL, He L, Perrimon N, Charras G, Giniger E, Baum B (2019). A role for actomyosin contractility in Notch signaling. BMC Biol.

[28] Wang Q, Lu Q (2017). Plasma membrane-derived extracellular microvesicles mediate non-canonical intercellular NOTCH signaling. Nat Commun.

[29] Sun Z, Wang L, Zhou Y, Dong L, Ma W, Lv L, Zhang J, Wang X (2020). Glioblastoma stem cell-derived exosomes enhance stemness and tumorigenicity of glioma cells by transferring Notch1 protein. Cell Mol Neurobiol.

[30] Borggrefe T, Oswald F (2009). The Notch signaling pathway: transcriptional regulation at Notch target genes. Cell Mol Life Sci.

[31] Wang H, Zang C, Liu XS, Aster JC (2015). The role of Notch receptors in transcriptional regulation. J Cell Physiol.

[32] Gomez-Lamarca MJ, Falo-Sanjuan J, Stojnic R, Abdul Rehman S, Muresan L, Jones ML, Pillidge Z, Cerda-Moya G, Yuan Z, Baloul S, Valenti P, Bystricky K, Payre F, O'Holleran K, Kovall R, Bray SJ (2018). Activation of the Notch signaling pathway in vivo elicits changes in CSL nuclear dynamics. Dev Cell..

[33] Kageyama R, Ohtsuka T, Kobayashi T (2007). The Hes gene family: repressors and oscillators that orchestrate embryogenesis. Development.

[34] Antfolk D, Antila C, Kemppainen K, Landor SK, Sahlgren C (2019). Decoding the PTM-switchboard of Notch. Biochim Biophys Acta Mol Cell Res.

[35] Chitnis A, Bally-Cuif L (2016). The Notch meeting: an odyssey from structure to function. Development.

[36] Lopez-Arribillaga E, Rodilla V, Colomer C, Vert A, Shelton A, Cheng JH, Yan B, Gonzalez-Perez A, Junttila MR, Iglesias M, Torres F, Albanell J, Villanueva A, Bigas A, Siebel CW, Espinosa L (2018). Manic fringe deficiency imposes Jagged1 addiction to intestinal tumor cells. Nat Commun.

[37] Wu G, Lyapina S, Das I, Li J, Gurney M, Pauley A, Chui I, Deshaies RJ, Kitajewski J (2001). SEL-10 is an inhibitor of notch signaling that targets notch for ubiquitin-mediated protein degradation. Mol Cell Biol.

[38] Morrugares R, Correa-Saez A, Moreno R, Garrido-Rodriguez M, Munoz E, de la Vega L, Calzado MA (2020). Phosphorylation-dependent regulation of the NOTCH1 intracellular domain by dual-specificity tyrosine-regulated kinase 2. Cell Mol Life Sci.

[39] Fryer CJ, White JB, Jones KA (2004). Mastermind recruits CycC:CDK8 to phosphorylate the Notch ICD and coordinate activation with turnover. Mol Cell.

[40] Hashimi ST, Fulcher JA, Chang MH, Gov L, Wang S, Lee B (2009). MicroRNA profiling identifies miR-34a and miR-21 and their target genes JAG1 and WNT1 in the coordinate regulation of dendritic cell differentiation. Blood.

[41] Li Y, Guessous F, Zhang Y, Dipierro C, Kefas B, Johnson E, Marcinkiewicz L, Jiang J, Yang Y, Schmittgen TD, Lopes B, Schiff D, Purow B, Abounader R (2009). MicroRNA-34a inhibits glioblastoma growth by targeting multiple oncogenes. Cancer Res.

[42] Yang M, Li CJ, Sun X, Guo Q, Xiao Y, Su T, Tu ML, Peng H, Lu Q, Liu Q, He HB, Jiang TJ, Lei MX, Wan M, Cao X, Luo XH (2017). MiR-497 approximately 195 cluster regulates angiogenesis during coupling with osteogenesis by maintaining endothelial Notch and HIF-1alpha activity. Nat Commun.

[43] Genz B, Coleman MA, Irvine KM, Kutasovic JR, Miranda M, Gratte FD, Tirnitz-Parker JEE, Olynyk JK, Calvopina DA, Weis A, Cloonan N, Robinson H, Hill MM, Al-Ejeh F, Ramm GA (2019). Overexpression of miRNA-25-3p inhibits Notch1 signaling and TGF-beta-induced collagen expression in hepatic stellate cells. Sci Rep.

[44] Zhao S, Xiao X, Sun S, Li D, Wang W, Fu Y, Fan F (2018). MicroRNA-30d/JAG1 axis modulates pulmonary fibrosis through Notch signaling pathway. Pathol Res Pract.

[45] Condorelli AG, Logli E, Cianfarani F, Teson M, Diociaiuti A, El Hachem M, Zambruno G, Castiglia D, Odorisio T (2019). MicroRNA-145-5p regulates fibrotic features of recessive dystrophic epidermolysis bullosa skin fibroblasts. Br J Dermatol.

[46] Li J, Dong S, Ye M, Peng G, Luo J, Wang C, Wang J, Zhao Q, Chang Y, Wang H (2020). MicroRNA-489-3p represses hepatic stellate cells activation by negatively regulating the JAG1/Notch3 signaling pathway. Dig Dis Sci.

[47] Wasson CW, Abignano G, Hermes H, Malaab M, Ross RL, Jimenez SA, Chang HY, Feghali-Bostwick CA, Del Galdo F (2020). Long non-coding RNA HOTAIR drives EZH2-dependent myofibroblast activation in systemic sclerosis through miRNA 34a-dependent activation of NOTCH. Ann Rheum Dis.

[48] Boucher JM, Peterson SM, Urs S, Zhang C, Liaw L (2011). The miR-143/145 cluster is a novel transcriptional target of Jagged-1/Notch signaling in vascular smooth muscle cells. J Biol Chem.

[49] Chandiran K, Lawlor R, Pannuti A, Perez GG, Srinivasan J, Golde TE, Miele L, Osborne BA, Minter LM (2018). Notch1 primes CD4 T cells for T helper type I differentiation through its early effects on miR-29. Mol Immunol.

[50] Kopp F, Mendell JT (2018). Functional classification and experimental dissection of long noncoding RNAs. Cell.

[51] Wan Y, Yang ZQ (2020). LncRNA NEAT1 affects inflammatory response by targeting miR-129-5p and regulating Notch signaling pathway in epilepsy. Cell Cycle.

[52] Rani N, Nowakowski TJ, Zhou H, Godshalk SE, Lisi V, Kriegstein AR, Kosik KS (2016). A primate lncRNA mediates Notch signaling during neuronal development by sequestering miRNA. Neuron.

[53] Trimarchi T, Bilal E, Ntziachristos P, Fabbri G, Dalla-Favera R, Tsirigos A, Aifantis I (2014). Genome-wide mapping and characterization of Notch-regulated long noncoding RNAs in acute leukemia. Cell.

[54] Zhang Z, Li G, Qiu H, Yang J, Bu X, Zhu S, Zheng J, Dang C, Wang W, Chu D (2019). The novel Notch-induced long noncoding RNA LUNAR1 determines the proliferation and prognosis of colorectal cancer. Sci Rep.

[55] Reicher A, Fosselteder J, Kwong LN, Pichler M (2018). Crosstalk between the Notch signaling pathway and long non-coding RNAs. Cancer Lett.

[56] Ghafouri-Fard S, Glassy MC, Abak A, Hussen BM, Niazi V, Taheri M (2021). The interaction between miRNAs/lncRNAs and Notch pathway in human disorders. Biomed Pharmacother.

[57] Tang Y, Boucher JM, Liaw L (2012). Histone deacetylase activity selectively regulates notch-mediated smooth muscle differentiation in human vascular cells. J Am Heart Assoc.

[58] Wang J, Wang CD, Zhang N, Tong WX, Zhang YF, Shan SZ, Zhang XL, Li QF (2016). Mechanical stimulation orchestrates the osteogenic differentiation of human bone marrow stromal cells by regulating HDAC1. Cell Death Dis.

[59] Tung CW, Hsu YC, Cai CJ, Shih YH, Wang CJ, Chang PJ, Lin CL (2017). Trichostatin A ameliorates renal tubulointerstitial fibrosis through modulation of the JNK-dependent Notch-2 signaling pathway. Sci Rep.

[60] Pinazza M, Ghisi M, Minuzzo S, Agnusdei V, Fossati G, Ciminale V, Pezze L, Ciribilli Y, Pilotto G, Venturoli C, Amadori A, Indraccolo S (2018). Histone deacetylase 6 controls Notch3 trafficking and degradation in T-cell acute lymphoblastic leukemia cells. Oncogene.

[61] Zhang C, Chen Y, Sun B, Wang L, Yang Y, Ma D, Lv J, Heng J, Ding Y, Xue Y, Lu X, Xiao W, Yang YG, Liu F (2017). m(6)A modulates haematopoietic stem and progenitor cell specification. Nature.

[62] Visvanathan A, Patil V, Abdulla S, Hoheisel JD, Somasundaram K (2019). N(6)-methyladenosine landscape of glioma stem-like cells: METTL3 is essential for the expression of actively transcribed genes and sustenance of the oncogenic signaling. Genes (Basel)..

[63] Maity A, Das B (2016). N6-methyladenosine modification in mRNA: machinery, function and implications for health and diseases. FEBS J.

[64] Warda AS, Kretschmer J, Hackert P, Lenz C, Urlaub H, Hobartner C, Sloan KE, Bohnsack MT (2017). Human METTL16 is a N(6)-methyladenosine (m(6)A) methyltransferase that targets pre-mRNAs and various non-coding RNAs. EMBO Rep..

[65] Mathiyalagan P, Adamiak M, Mayourian J, Sassi Y, Liang Y, Agarwal N, Jha D, Zhang S, Kohlbrenner E, Chepurko E, Chen J, Trivieri MG, Singh R, Bouchareb R, Fish K, Ishikawa K, Lebeche D, Hajjar RJ, Sahoo S (2019). FTO-Dependent N(6)-methyladenosine regulates cardiac function during remodeling and repair. Circulation.

[66] Huang H, Weng H, Chen J (2020). m(6)A modification in coding and non-coding RNAs: roles and therapeutic implications in cancer. Cancer Cell.

[67] Schaefer L (2018). Decoding fibrosis: mechanisms and translational aspects. Matrix Biol.

[68] Landen NX, Li D, Stahle M (2016). Transition from inflammation to proliferation: a critical step during wound healing. Cell Mol Life Sci.

[69] Cianfarani F, Zambruno G, Castiglia D, Odorisio T (2017). Pathomechanisms of altered wound healing in recessive dystrophic epidermolysis bullosa. Am J Pathol.

[70] Pakshir P, Hinz B (2018). The big five in fibrosis: macrophages, myofibroblasts, matrix, mechanics, and miscommunication. Matrix Biol.

[71] Hinz B, McCulloch CA, Coelho NM (2019). Mechanical regulation of myofibroblast phenoconversion and collagen contraction. Exp Cell Res.

[72] Huang S, Park J, Qiu C, Chung KW, Li SY, Sirin Y, Han SH, Taylor V, Zimber-Strobl U, Susztak K (2018). Jagged1/Notch2 controls kidney fibrosis via Tfam-mediated metabolic reprogramming. PLoS Biol.

[73] Zhu C, Kim K, Wang X, Bartolome A, Salomao M, Dongiovanni P, Meroni M, Graham MJ, Yates KP, Diehl AM, Schwabe RF, Tabas I, Valenti L, Lavine JE, Pajvani UB (2018). Hepatocyte Notch activation induces liver fibrosis in nonalcoholic steatohepatitis. Sci Transl Med..

[74] Wang YC, Chen Q, Luo JM, Nie J, Meng QH, Shuai W, Xie H, Xia JM, Wang H (2019). Notch1 promotes the pericyte-myofibroblast transition in idiopathic pulmonary fibrosis through the PDGFR/ROCK1 signal pathway. Exp Mol Med.

[75] Cereseto A, Tsai S (2000). Jagged2 induces cell cycling in confluent fibroblasts susceptible to density-dependent inhibition of cell division. J Cell Physiol.

[76] Dees C, Tomcik M, Zerr P, Akhmetshina A, Horn A, Palumbo K, Beyer C, Zwerina J, Distler O, Schett G, Distler JH (2011). Notch signalling regulates fibroblast activation and collagen release in systemic sclerosis. Ann Rheum Dis.

[77] Doi H, Iso T, Sato H, Yamazaki M, Matsui H, Tanaka T, Manabe I, Arai M, Nagai R, Kurabayashi M (2006). Jagged1-selective notch signaling induces smooth muscle differentiation via a RBP-Jkappa-dependent pathway. J Biol Chem..

[78] Bhattacharyya A, Lin S, Sandig M, Mequanint K (2014). Regulation of vascular smooth muscle cell phenotype in three-dimensional coculture system by Jagged1-selective Notch3 signaling. Tissue Eng Part A.

[79] Klose R, Prinz A, Tetzlaff F, Weis EM, Moll I, Rodriguez-Vita J, Oka C, Korff T, Fischer A (2019). Loss of the serine protease HTRA1 impairs smooth muscle cells maturation. Sci Rep.

[80] Noseda M, McLean G, Niessen K, Chang L, Pollet I, Montpetit R, Shahidi R, Dorovini-Zis K, Li L, Beckstead B, Durand RE, Hoodless PA, Karsan A (2004). Notch activation results in phenotypic and functional changes consistent with endothelial-to-mesenchymal transformation. Circ Res.

[81] Noseda M, Fu Y, Niessen K, Wong F, Chang L, McLean G, Karsan A (2006). Smooth muscle alpha-actin is a direct target of Notch/CSL. Circ Res.

[82] Tang Y, Urs S, Liaw L (2008). Hairy-related transcription factors inhibit Notch-induced smooth muscle alpha-actin expression by interfering with Notch intracellular domain/CBF-1 complex interaction with the CBF-1-binding site. Circ Res.

[83] Zavadil J, Cermak L, Soto-Nieves N, Bottinger EP (2004). Integration of TGF-beta/Smad and Jagged1/Notch signalling in epithelial-to-mesenchymal transition. EMBO J.

[84] Radtke F, Fasnacht N, Macdonald HR (2010). Notch signaling in the immune system. Immunity.

[85] Wei K, Korsunsky I, Marshall JL, Gao A, Watts GFM, Major T, Croft AP, Watts J, Blazar PE, Lange JK, Thornhill TS, Filer A, Raza K, Donlin LT, Accelerating Medicines Partnership Rheumatoid A, Systemic Lupus Erythematosus C, Siebel CW, Buckley CD, Raychaudhuri S, Brenner MB. Notch signalling drives synovial fibroblast identity and arthritis pathology. Nature. 2020;582(7811):259–64. 10.1038/s41586-020-2222-z. 10.1038/s41586-020-2222-z PMC7841716 32499639

[86] Zhao X, Psarianos P, Ghoraie LS, Yip K, Goldstein D, Gilbert R, Witterick I, Pang H, Hussain A, Lee JH, Williams J, Bratman SV, Ailles L, Haibe-Kains B, Liu FF (2019). Metabolic regulation of dermal fibroblasts contributes to skin extracellular matrix homeostasis and fibrosis. Nat Metab.

[87] de Castro Bras LE, Frangogiannis NG (2020). Extracellular matrix-derived peptides in tissue remodeling and fibrosis. Matrix Biol.

[88] DeLeon-Pennell KY, Barker TH, Lindsey ML (2020). Fibroblasts: the arbiters of extracellular matrix remodeling. Matrix Biol.

[89] Frantz C, Stewart KM, Weaver VM (2010). The extracellular matrix at a glance. J Cell Sci.

[90] Nystrom A, Bruckner-Tuderman L (2019). Matrix molecules and skin biology. Semin Cell Dev Biol.

[91] Iozzo RV, Gubbiotti MA (2018). Extracellular matrix: the driving force of mammalian diseases. Matrix Biol.

[92] Nystrom A, Bernasconi R, Bornert O (2018). Therapies for genetic extracellular matrix diseases of the skin. Matrix Biol.

[93] Bhattacharjee O, Ayyangar U, Kurbet AS, Ashok D, Raghavan S (2019). Unraveling the ECM-immune cell crosstalk in skin diseases. Front Cell Dev Biol.

[94] Herrera J, Henke CA, Bitterman PB (2018). Extracellular matrix as a driver of progressive fibrosis. J Clin Invest.

[95] Ricard-Blum S, Baffet G, Theret N (2018). Molecular and tissue alterations of collagens in fibrosis. Matrix Biol.

[96] Henke E, Nandigama R, Ergun S (2019). Extracellular matrix in the tumor microenvironment and its impact on cancer therapy. Front Mol Biosci.

[97] Hinz B (2015). The extracellular matrix and transforming growth factor-beta1: tale of a strained relationship. Matrix Biol.

[98] Schulz JN, Plomann M, Sengle G, Gullberg D, Krieg T, Eckes B (2018). New developments on skin fibrosis—essential signals emanating from the extracellular matrix for the control of myofibroblasts. Matrix Biol.

[99] Schnieder J, Mamazhakypov A, Birnhuber A, Wilhelm J, Kwapiszewska G, Ruppert C, Markart P, Wujak L, Rubio K, Barreto G, Schaefer L, Wygrecka M (2020). Loss of LRP1 promotes acquisition of contractile-myofibroblast phenotype and release of active TGF-beta1 from ECM stores. Matrix Biol.

[100] Kechagia JZ, Ivaska J, Roca-Cusachs P (2019). Integrins as biomechanical sensors of the microenvironment. Nat Rev Mol Cell Biol.

[101] LaFoya B, Munroe JA, Mia MM, Detweiler MA, Crow JJ, Wood T, Roth S, Sharma B, Albig AR (2016). Notch: a multi-functional integrating system of microenvironmental signals. Dev Biol.

[102] Lemaire R, Korn JH, Shipley JM, Lafyatis R (2005). Increased expression of type I collagen induced by microfibril-associated glycoprotein 2: novel mechanistic insights into the molecular basis of dermal fibrosis in scleroderma. Arthritis Rheum.

[103] Nehring LC, Miyamoto A, Hein PW, Weinmaster G, Shipley JM (2005). The extracellular matrix protein MAGP-2 interacts with Jagged1 and induces its shedding from the cell surface. J Biol Chem.

[104] Miyamoto A, Lau R, Hein PW, Shipley JM, Weinmaster G (2006). Microfibrillar proteins MAGP-1 and MAGP-2 induce Notch1 extracellular domain dissociation and receptor activation. J Biol Chem.

[105] Deford P, Brown K, Richards RL, King A, Newburn K, Westover K, Albig AR (2016). MAGP2 controls Notch via interactions with RGD binding integrins: identification of a novel ECM-integrin-Notch signaling axis. Exp Cell Res.

[106] LaFoya B, Munroe JA, Pu X, Albig AR (2018). Src kinase phosphorylates Notch1 to inhibit MAML binding. Sci Rep.

[107] Tanabe H, Takayama I, Nishiyama T, Shimazaki M, Kii I, Li M, Amizuka N, Katsube K, Kudo A (2010). Periostin associates with Notch1 precursor to maintain Notch1 expression under a stress condition in mouse cells. PLoS ONE.

[108] Zhang X, Meng H, Wang MM (2013). Collagen represses canonical Notch signaling and binds to Notch ectodomain. Int J Biochem Cell Biol.

[109] Estrach S, Cailleteau L, Franco CA, Gerhardt H, Stefani C, Lemichez E, Gagnoux-Palacios L, Meneguzzi G, Mettouchi A (2011). Laminin-binding integrins induce Dll4 expression and Notch signaling in endothelial cells. Circ Res.

[110] David CJ, Massague J (2018). Contextual determinants of TGFbeta action in development, immunity and cancer. Nat Rev Mol Cell Biol.

[111] Massague J (2000). How cells read TGF-beta signals. Nat Rev Mol Cell Biol.

[112] Shi Y, Massague J (2003). Mechanisms of TGF-beta signaling from cell membrane to the nucleus. Cell.

[113] Frangogiannis N (2020). Transforming growth factor-beta in tissue fibrosis. J Exp Med.

[114] Lodyga M, Hinz B (2020). TGF-beta1—a truly transforming growth factor in fibrosis and immunity. Semin Cell Dev Biol.

[115] Zavadil J, Bitzer M, Liang D, Yang YC, Massimi A, Kneitz S, Piek E, Bottinger EP (2001). Genetic programs of epithelial cell plasticity directed by transforming growth factor-beta. Proc Natl Acad Sci U S A.

[116] Kluppel M, Wrana JL (2005). Turning it up a Notch: cross-talk between TGF beta and Notch signaling. BioEssays.

[117] Grieskamp T, Rudat C, Ludtke TH, Norden J, Kispert A (2011). Notch signaling regulates smooth muscle differentiation of epicardium-derived cells. Circ Res.

[118] Ohnuki H, Jiang K, Wang D, Salvucci O, Kwak H, Sanchez-Martin D, Maric D, Tosato G (2014). Tumor-infiltrating myeloid cells activate Dll4/Notch/TGF-beta signaling to drive malignant progression. Cancer Res.

[119] Borggrefe T, Lauth M, Zwijsen A, Huylebroeck D, Oswald F, Giaimo BD (2016). The Notch intracellular domain integrates signals from Wnt, Hedgehog, TGFbeta/BMP and hypoxia pathways. Biochim Biophys Acta.

[120] Blokzijl A, Dahlqvist C, Reissmann E, Falk A, Moliner A, Lendahl U, Ibanez CF (2003). Cross-talk between the Notch and TGF-beta signaling pathways mediated by interaction of the Notch intracellular domain with Smad3. J Cell Biol.

[121] Xiao Z, Zhang J, Peng X, Dong Y, Jia L, Li H, Du J (2014). The Notch gamma-secretase inhibitor ameliorates kidney fibrosis via inhibition of TGF-beta/Smad2/3 signaling pathway activation. Int J Biochem Cell Biol.

[122] Liu Y, Huang G, Mo B, Wang C (2017). Artesunate ameliorates lung fibrosis via inhibiting the Notch signaling pathway. Exp Ther Med.

[123] Wang Y, Shen RW, Han B, Li Z, Xiong L, Zhang FY, Cong BB, Zhang B (2017). Notch signaling mediated by TGF-beta/Smad pathway in concanavalin A-induced liver fibrosis in rats. World J Gastroenterol.

[124] Fan J, Shen W, Lee SR, Mathai AE, Zhang R, Xu G, Gillies MC (2020). Targeting the Notch and TGF-beta signaling pathways to prevent retinal fibrosis in vitro and in vivo. Theranostics.

[125] Tang Y, Urs S, Boucher J, Bernaiche T, Venkatesh D, Spicer DB, Vary CP, Liaw L (2010). Notch and transforming growth factor-beta (TGFbeta) signaling pathways cooperatively regulate vascular smooth muscle cell differentiation. J Biol Chem.

[126] Kurpinski K, Lam H, Chu J, Wang A, Kim A, Tsay E, Agrawal S, Schaffer DV, Li S (2010). Transforming growth factor-beta and notch signaling mediate stem cell differentiation into smooth muscle cells. Stem Cells.

[127] Aoyagi-Ikeda K, Maeno T, Matsui H, Ueno M, Hara K, Aoki Y, Aoki F, Shimizu T, Doi H, Kawai-Kowase K, Iso T, Suga T, Arai M, Kurabayashi M (2011). Notch induces myofibroblast differentiation of alveolar epithelial cells via transforming growth factor-{beta}-Smad3 pathway. Am J Respir Cell Mol Biol..

[128] Manokawinchoke J, Sumrejkanchanakij P, Pavasant P, Osathanon T (2017). Notch signaling participates in TGF-beta-induced SOST expression under intermittent compressive stress. J Cell Physiol.

[129] Piersma B, de Rond S, Werker PM, Boo S, Hinz B, van Beuge MM, Bank RA (2015). YAP1 is a driver of myofibroblast differentiation in normal and diseased fibroblasts. Am J Pathol.

[130] Totaro A, Castellan M, Di Biagio D, Piccolo S (2018). Crosstalk between YAP/TAZ and Notch signaling. Trends Cell Biol.

[131] Burgy O, Konigshoff M (2018). The WNT signaling pathways in wound healing and fibrosis. Matrix Biol.

[132] Foltz DR, Santiago MC, Berechid BE, Nye JS (2002). Glycogen synthase kinase-3beta modulates notch signaling and stability. Curr Biol.

[133] Espinosa L, Ingles-Esteve J, Aguilera C, Bigas A (2003). Phosphorylation by glycogen synthase kinase-3 beta down-regulates Notch activity, a link for Notch and Wnt pathways. J Biol Chem.

[134] Jin YH, Kim H, Ki H, Yang I, Yang N, Lee KY, Kim N, Park HS, Kim K (2009). Beta-catenin modulates the level and transcriptional activity of Notch1/NICD through its direct interaction. Biochim Biophys Acta.

[135] Peignon G, Durand A, Cacheux W, Ayrault O, Terris B, Laurent-Puig P, Shroyer NF, Van Seuningen I, Honjo T, Perret C, Romagnolo B (2011). Complex interplay between beta-catenin signalling and Notch effectors in intestinal tumorigenesis. Gut.

[136] Collu GM, Hidalgo-Sastre A, Brennan K (2014). Wnt-Notch signalling crosstalk in development and disease. Cell Mol Life Sci.

[137] Zheng L, Conner SD (2018). Glycogen synthase kinase 3beta inhibition enhances Notch1 recycling. Mol Biol Cell.

[138] Edeling M, Ragi G, Huang S, Pavenstadt H, Susztak K (2016). Developmental signalling pathways in renal fibrosis: the roles of Notch, Wnt and Hedgehog. Nat Rev Nephrol.

[139] Thelu J, Rossio P, Favier B (2002). Notch signalling is linked to epidermal cell differentiation level in basal cell carcinoma, psoriasis and wound healing. BMC Dermatol.

[140] Blanpain C, Lowry WE, Pasolli HA, Fuchs E (2006). Canonical notch signaling functions as a commitment switch in the epidermal lineage. Genes Dev.

[141] Okuyama R, Tagami H, Aiba S (2008). Notch signaling: its role in epidermal homeostasis and in the pathogenesis of skin diseases. J Dermatol Sci.

[142] Nowell C, Radtke F (2013). Cutaneous Notch signaling in health and disease. Cold Spring Harb Perspect Med.

[143] Lowell S, Jones P, Le Roux I, Dunne J, Watt FM (2000). Stimulation of human epidermal differentiation by delta-notch signalling at the boundaries of stem-cell clusters. Curr Biol.

[144] Nickoloff BJ, Qin JZ, Chaturvedi V, Denning MF, Bonish B, Miele L (2002). Jagged-1 mediated activation of notch signaling induces complete maturation of human keratinocytes through NF-kappaB and PPARgamma. Cell Death Differ.

[145] Negri VA, Logtenberg MEW, Renz LM, Oules B, Walko G, Watt FM (2019). Delta-like 1-mediated cis-inhibition of Jagged1/2 signalling inhibits differentiation of human epidermal cells in culture. Sci Rep.

[146] Nicolas M, Wolfer A, Raj K, Kummer JA, Mill P, van Noort M, Hui CC, Clevers H, Dotto GP, Radtke F (2003). Notch1 functions as a tumor suppressor in mouse skin. Nat Genet.

[147] Wang NJ, Sanborn Z, Arnett KL, Bayston LJ, Liao W, Proby CM, Leigh IM, Collisson EA, Gordon PB, Jakkula L, Pennypacker S, Zou Y, Sharma M, North JP, Vemula SS, Mauro TM, Neuhaus IM, Leboit PE, Hur JS, Park K, Huh N, Kwok PY, Arron ST, Massion PP, Bale AE, Haussler D, Cleaver JE, Gray JW, Spellman PT, South AP, Aster JC, Blacklow SC, Cho RJ (2011). Loss-of-function mutations in Notch receptors in cutaneous and lung squamous cell carcinoma. Proc Natl Acad Sci U S A.

[148] Gratton R, Tricarico PM, Moltrasio C, Lima Estevao de Oliveira AS, Brandao L, Marzano AV, Zupin L, Crovella S (2020). Pleiotropic role of Notch signaling in human skin diseases. Int J Mol Sci..

[149] Kim JE, Lee JH, Jeong KH, Kim GM, Kang H (2014). Notch intracellular domain expression in various skin fibroproliferative diseases. Ann Dermatol.

[150] Abdulle AE, Diercks GFH, Feelisch M, Mulder DJ, van Goor H (2018). The role of oxidative stress in the development of systemic sclerosis related vasculopathy. Front Physiol.

[151] Krieg T, Takehara K (2009). Skin disease: a cardinal feature of systemic sclerosis. Rheumatology (Oxford).

[152] Ayers NB, Sun CM, Chen SY (2018). Transforming growth factor-beta signaling in systemic sclerosis. J Biomed Res.

[153] Zmorzynski S, Styk W, Filip AA, Krasowska D (2019). The significance of NOTCH pathway in the development of fibrosis in systemic sclerosis. Ann Dermatol.

[154] Kavian N, Servettaz A, Mongaret C, Wang A, Nicco C, Chereau C, Grange P, Vuiblet V, Birembaut P, Diebold MD, Weill B, Dupin N, Batteux F (2010). Targeting ADAM-17/notch signaling abrogates the development of systemic sclerosis in a murine model. Arthritis Rheum.

[155] Zhang Z, Oliver P, Lancaster JR, Schwarzenberger PO, Joshi MS, Cork J, Kolls JK (2001). Reactive oxygen species mediate tumor necrosis factor alpha-converting, enzyme-dependent ectodomain shedding induced by phorbol myristate acetate. FASEB J.

[156] Shao MX, Nadel JA (2005). Dual oxidase 1-dependent MUC5AC mucin expression in cultured human airway epithelial cells. Proc Natl Acad Sci U S A.

[157] Valadi H, Ekstrom K, Bossios A, Sjostrand M, Lee JJ, Lotvall JO (2007). Exosome-mediated transfer of mRNAs and microRNAs is a novel mechanism of genetic exchange between cells. Nat Cell Biol.

[158] Colletti M, Galardi A, De Santis M, Guidelli GM, Di Giannatale A, Di Luigi L, Antinozzi C (2019). Exosomes in systemic sclerosis: messengers between immune, vascular and fibrotic components?. Int J Mol Sci..

[159] Wermuth PJ, Piera-Velazquez S, Jimenez SA (2017). Exosomes isolated from serum of systemic sclerosis patients display alterations in their content of profibrotic and antifibrotic microRNA and induce a profibrotic phenotype in cultured normal dermal fibroblasts. Clin Exp Rheumatol..

[160] Li L, Zuo X, Liu D, Luo H, Zhu H (2020). The profiles of miRNAs and lncRNAs in peripheral blood neutrophils exosomes of diffuse cutaneous systemic sclerosis. J Dermatol Sci.

[161] Iwamoto N, Distler JH, Distler O (2011). Tyrosine kinase inhibitors in the treatment of systemic sclerosis: from animal models to clinical trials. Curr Rheumatol Rep.

[162] Fraticelli P, Gabrielli B, Pomponio G, Valentini G, Bosello S, Riboldi P, Gerosa M, Faggioli P, Giacomelli R, Del Papa N, Gerli R, Lunardi C, Bombardieri S, Malorni W, Corvetta A, Moroncini G, Gabrielli A, Imatinib in Scleroderma Italian Study G (2014). Low-dose oral imatinib in the treatment of systemic sclerosis interstitial lung disease unresponsive to cyclophosphamide: a phase II pilot study. Arthritis Res Ther..

[163] Harrach S, Barz V, Pap T, Pavenstadt H, Schlatter E, Edemir B, Distler J, Ciarimboli G, Bertrand J (2019). Notch signaling activity determines uptake and biological effect of imatinib in systemic sclerosis dermal fibroblasts. J Invest Dermatol.

[164] Zmorzynski S, Wojcierowska-Litwin M, Kowal M, Michalska-Jakubus M, Styk W, Filip AA, Walecka I, Krasowska D (2020). NOTCH3 T6746C and TP53 P72R polymorphisms are associated with the susceptibility to diffuse cutaneous systemic sclerosis. Biomed Res Int.

[165] Berman B, Maderal A, Raphael B (2017). Keloids and hypertrophic scars: pathophysiology, classification, and treatment. Dermatol Surg.

[166] Limandjaja GC, Belien JM, Scheper RJ, Niessen FB, Gibbs S (2020). Hypertrophic and keloid scars fail to progress from the CD34(−) /alpha-smooth muscle actin (alpha-SMA)(+) immature scar phenotype and show gradient differences in alpha-SMA and p16 expression. Br J Dermatol.

[167] Machesney M, Tidman N, Waseem A, Kirby L, Leigh I (1998). Activated keratinocytes in the epidermis of hypertrophic scars. Am J Pathol.

[168] Li B, Gao C, Diao JS, Wang DL, Chu FF, Li Y, Wang G, Guo SZ, Xia W (2016). Aberrant Notch signalling contributes to hypertrophic scar formation by modulating the phenotype of keratinocytes. Exp Dermatol.

[169] Zhao J, Yu J, Xu Y, Chen L, Zhou F, Zhai Q, Wu J, Shu B, Qi S (2018). Epidermal HMGB1 activates dermal fibroblasts and causes hypertrophic scar formation in reduced hydration. J Invest Dermatol.

[170] Feng Y, Sun ZL, Liu SY, Wu JJ, Zhao BH, Lv GZ, Du Y, Yu S, Yang ML, Yuan FL, Zhou XJ (2019). Direct and indirect roles of macrophages in hypertrophic scar formation. Front Physiol.

[171] He T, Bai X, Jing J, Liu Y, Wang H, Zhang W, Li X, Li Y, Wang L, Xie S, Hu D (2020). Notch signal deficiency alleviates hypertrophic scar formation after wound healing through the inhibition of inflammation. Arch Biochem Biophys.

[172] Han B, Fan J, Liu L, Tian J, Gan C, Yang Z, Jiao H, Zhang T, Liu Z, Zhang H (2019). Adipose-derived mesenchymal stem cells treatments for fibroblasts of fibrotic scar via downregulating TGF-beta1 and Notch-1 expression enhanced by photobiomodulation therapy. Lasers Med Sci.

[173] Andrews JP, Marttala J, Macarak E, Rosenbloom J, Uitto J (2016). Keloids: the paradigm of skin fibrosis—pathomechanisms and treatment. Matrix Biol.

[174] Syed F, Bayat A (2012). Notch signaling pathway in keloid disease: enhanced fibroblast activity in a Jagged-1 peptide-dependent manner in lesional vs extralesional fibroblasts. Wound Repair Regen..

[175] Lee S, Kim SK, Park H, Lee YJ, Park SH, Lee KJ, Lee DG, Kang H, Kim JE (2020). Contribution of autophagy-Notch1-mediated NLRP3 inflammasome activation to chronic inflammation and fibrosis in keloid fibroblasts. Int J Mol Sci..

[176] Onoufriadis A, Hsu CK, Ainali C, Ung CY, Rashidghamat E, Yang HS, Huang HY, Niazi U, Tziotzios C, Yang JC, Nuamah R, Tang MJ, Saxena A, de Rinaldis E, McGrath JA (2018). Time series integrative analysis of RNA sequencing and microRNA expression data reveals key biologic wound healing pathways in keloid-prone individuals. J Invest Dermatol.

[177] Has C, Bauer JW, Bodemer C, Bolling MC, Bruckner-Tuderman L, Diem A, Fine JD, Heagerty A, Hovnanian A, Marinkovich MP, Martinez AE, McGrath JA, Moss C, Murrell DF, Palisson F, Schwieger-Briel A, Sprecher E, Tamai K, Uitto J, Woodley DT, Zambruno G, Mellerio JE (2020). Consensus reclassification of inherited epidermolysis bullosa and other disorders with skin fragility. Br J Dermatol.

[178] Odorisio T, Di Salvio M, Orecchia A, Di Zenzo G, Piccinni E, Cianfarani F, Travaglione A, Uva P, Bellei B, Conti A, Zambruno G, Castiglia D (2014). Monozygotic twins discordant for recessive dystrophic epidermolysis bullosa phenotype highlight the role of TGF-beta signalling in modifying disease severity. Hum Mol Genet.

[179] Guerra L, Odorisio T, Zambruno G, Castiglia D (2017). Stromal microenvironment in type VII collagen-deficient skin: the ground for squamous cell carcinoma development. Matrix Biol.

[180] Condorelli AG, Dellambra E, Logli E, Zambruno G, Castiglia D (2019). Epidermolysis bullosa-associated squamous cell carcinoma: from pathogenesis to therapeutic perspectives. Int J Mol Sci..

[181] Cho RJ, Alexandrov LB, den Breems NY, Atanasova VS, Farshchian M, Purdom E, Nguyen TN, Coarfa C, Rajapakshe K, Prisco M, Sahu J, Tassone P, Greenawalt EJ, Collisson EA, Wu W, Yao H, Su X, Guttmann-Gruber C, Hofbauer JP, Hashmi R, Fuentes I, Benz SC, Golovato J, Ehli EA, Davis CM, Davies GE, Covington KR, Murrell DF, Salas-Alanis JC, Palisson F, Bruckner AL, Robinson W, Has C, Bruckner-Tuderman L, Titeux M, Jonkman MF, Rashidghamat E, Lwin SM, Mellerio JE, McGrath JA, Bauer JW, Hovnanian A, Tsai KY, South AP (2018). APOBEC mutation drives early-onset squamous cell carcinomas in recessive dystrophic epidermolysis bullosa. Sci Transl Med..

[182] Massi D, Tarantini F, Franchi A, Paglierani M, Di Serio C, Pellerito S, Leoncini G, Cirino G, Geppetti P, Santucci M (2006). Evidence for differential expression of Notch receptors and their ligands in melanocytic nevi and cutaneous malignant melanoma. Mod Pathol.

[183] Kolev V, Mandinova A, Guinea-Viniegra J, Hu B, Lefort K, Lambertini C, Neel V, Dummer R, Wagner EF, Dotto GP (2008). EGFR signalling as a negative regulator of Notch1 gene transcription and function in proliferating keratinocytes and cancer. Nat Cell Biol.

[184] Al Labban D, Jo SH, Ostano P, Saglietti C, Bongiovanni M, Panizzon R, Dotto GP (2018). Notch-effector CSL promotes squamous cell carcinoma by repressing histone demethylase KDM6B. J Clin Invest.

[185] Andersson ER, Lendahl U (2014). Therapeutic modulation of Notch signalling—are we there yet?. Nat Rev Drug Discov.

[186] Yuan X, Wu H, Xu H, Xiong H, Chu Q, Yu S, Wu GS, Wu K (2015). Notch signaling: an emerging therapeutic target for cancer treatment. Cancer Lett.

[187] Katoh M, Katoh M (2020). Precision medicine for human cancers with Notch signaling dysregulation (Review). Int J Mol Med.

[188] Moore G, Annett S, McClements L, Robson T (2020). Top Notch targeting strategies in cancer: a detailed overview of recent insights and current perspectives. Cells.

[189] De Kloe GE, De Strooper B (2014). Small molecules that inhibit Notch signaling. Methods Mol Biol.

[190] Ntziachristos P, Lim JS, Sage J, Aifantis I (2014). From fly wings to targeted cancer therapies: a centennial for notch signaling. Cancer Cell.

[191] Ran Y, Hossain F, Pannuti A, Lessard CB, Ladd GZ, Jung JI, Minter LM, Osborne BA, Miele L, Golde TE (2017). gamma-Secretase inhibitors in cancer clinical trials are pharmacologically and functionally distinct. EMBO Mol Med..

[192] Massard C, Azaro A, Soria JC, Lassen U, Le Tourneau C, Sarker D, Smith C, Ohnmacht U, Oakley G, Patel BKR, Yuen ESM, Benhadji KA, Rodon J (2018). First-in-human study of LY3039478, an oral Notch signaling inhibitor in advanced or metastatic cancer. Ann Oncol.

[193] Hyde LA, McHugh NA, Chen J, Zhang Q, Manfra D, Nomeir AA, Josien H, Bara T, Clader JW, Zhang L, Parker EM, Higgins GA (2006). Studies to investigate the in vivo therapeutic window of the gamma-secretase inhibitor N2-[(2S)-2-(3,5-difluorophenyl)-2-hydroxyethanoyl]-N1-[(7S)-5-methyl-6-oxo-6,7-di hydro-5H-dibenzo[b, d]azepin-7-yl]-L-alaninamide (LY411,575) in the CRND8 mouse. J Pharmacol Exp Ther.

[194] Distler A, Lang V, Del Vecchio T, Huang J, Zhang Y, Beyer C, Lin NY, Palumbo-Zerr K, Distler O, Schett G, Distler JH (2014). Combined inhibition of morphogen pathways demonstrates additive antifibrotic effects and improved tolerability. Ann Rheum Dis.

[195] Moellering RE, Cornejo M, Davis TN, Del Bianco C, Aster JC, Blacklow SC, Kung AL, Gilliland DG, Verdine GL, Bradner JE (2009). Direct inhibition of the NOTCH transcription factor complex. Nature.

[196] Wu Y, Cain-Hom C, Choy L, Hagenbeek TJ, de Leon GP, Chen Y, Finkle D, Venook R, Wu X, Ridgway J, Schahin-Reed D, Dow GJ, Shelton A, Stawicki S, Watts RJ, Zhang J, Choy R, Howard P, Kadyk L, Yan M, Zha J, Callahan CA, Hymowitz SG, Siebel CW (2010). Therapeutic antibody targeting of individual Notch receptors. Nature.

[197] Astudillo L, Da Silva TG, Wang Z, Han X, Jin K, VanWye J, Zhu X, Weaver K, Oashi T, Lopes PE, Orton D, Neitzel LR, Lee E, Landgraf R, Robbins DJ, MacKerell AD, Capobianco AJ (2016). The small molecule IMR-1 inhibits the Notch transcriptional activation complex to suppress tumorigenesis. Cancer Res.

[198] Gomez-Galeno JE, Hurtado C, Cheng J, Yardimci C, Mercola M, Cashman JR (2018). b-Annulated 1,4-dihydropyridines as Notch inhibitors. Bioorg Med Chem Lett.

[199] Hurtado C, Safarova A, Smith M, Chung R, Bruyneel AAN, Gomez-Galeno J, Oswald F, Larson CJ, Cashman JR, Ruiz-Lozano P, Janiak P, Suzuki T, Mercola M (2019). Disruption of NOTCH signaling by a small molecule inhibitor of the transcription factor RBPJ. Sci Rep.

[200] Masiero M, Li D, Whiteman P, Bentley C, Greig J, Hassanali T, Watts S, Stribbling S, Yates J, Bealing E, Li JL, Chillakuri C, Sheppard D, Serres S, Sarmiento-Soto M, Larkin J, Sibson NR, Handford PA, Harris AL, Banham AH (2019). Development of therapeutic anti-JAGGED1 antibodies for cancer therapy. Mol Cancer Ther.

[201] Zhou Y, Liao S, Zhang Z, Wang B, Wan L (2016). Astragalus injection attenuates bleomycin-induced pulmonary fibrosis via down-regulating Jagged1/Notch1 in lungs. J Pharm Pharmacol.

