# Supplementary material for: Notch-ing up knowledge on molecular mechanisms of skin fibrosis: focus on the multifaceted Notch signalling pathway
Source: J Biomed Sci. 2021 May 9;28:36. doi: 10.1186/s12929-021-00732-8 (PMC8106838; doi:10.1186/s12929-021-00732-8)
Supplement: Supplementary file 1 — Additional file 1: Table S1. Luciferase reporter assay-validated miRNAs regulating Notch receptors and ligands. Table S2. Drugs and compounds targeting Notch pathway. [file 12929_2021_732_MOESM1_ESM.docx]

**Table S1. Luciferase reporter assay-validated miRNAs regulating Notch receptors and ligands.**

| **Target name** | **miRNA name** | **References** |
| --- | --- | --- |
| **NOTCH1** | hsa-miR-101-3p | [1] |
|  | hsa-miR-10b-5p | [2] |
|  | hsa-miR-139-5p | [3-5] |
|  | hsa-miR-146a-5p | [6] |
|  | hsa-miR-200b-3p | [7] |
|  | hsa-miR-200c-3p | [7] |
|  | hsa-miR-30a-3p | [8] |
|  | hsa-miR-30a-5p | [9] |
|  | hsa-miR-30b-5p | [9] |
|  | hsa-miR-30c-5p | [9] |
|  | hsa-miR-30d-5p | [9] |
|  | hsa-miR-30e-5p | [9] |
|  | hsa-miR-326 | [10] |
|  | hsa-miR-34a-5p | [11-19] |
|  | hsa-miR-34b-3p | [7,20] |
|  | hsa-miR-34c-5p | [7,20] |
|  | hsa-miR-363-3p | [21] |
|  | hsa-miR-449 | [22] |
|  | hsa-miR-935 | [23] |
|  | hsa-miR-9-5p | [24] |
| **NOTCH2** | hsa-miR-107 | [25] |
|  | hsa-miR-146a-5p | [6,26,27] |
|  | hsa-miR-191-5p | [28] |
|  | hsa-miR-23b-3p | [29] |
|  | hsa-miR-30a-3p | [8] |
|  | hsa-miR-326 | [10] |
|  | hsa-miR-34a-5p | [12,14] |
|  | hsa-miR-9-3p/-5p | [30] |
| **NOTCH3** | hsa-miR-136-3p | [31] |
|  | hsa-miR-1-3p | [32,33] |
|  | hsa-miR-150-5p | [34] |
|  | hsa-miR-206 | [32] |
|  | hsa-miR-483-5p | [35] |
|  | hsa-miR-491-5p | [36] |
| **NOTCH4** | hsa-miR-181c-5p | [37] |
|  | hsa-miR-34c-5p | [38] |
| **JAG1** | hsa-miR-124-3p | [39,40] |
|  | hsa-miR-199b-5p | [41,42] |
|  | hsa-miR-214-3p | [39] |
|  | hsa-miR-21-5p | [43,44] |
|  | hsa-miR-26a-5p | [45] |
|  | hsa-miR-26b-5p | [46] |
|  | hsa-miR-34a-5p | [14,43,47] |
|  | hsa-miR-34b-5p | [48] |
|  | hsa-miR-489-3p | [49] |
|  | hsa-miR-524-5p | [50] |
| **JAG2** | ND | ND |
| **DLL1** | hsa-miR-130b-3p | [51] |
|  | hsa-miR-34a-5p | [11,52,53] |
| **DLL3** | ND | ND |
| **DLL4** | ND | ND |

**Additional file 1: Table S1. Luciferase reporter assay-validated microRNAs regulating Notch receptors (NOTCH1-4) and ligands (JAG1-2, DLL1, DLL3-4).** Firstly we retrieved from miRTarBase (release 8.0) (http://miRTarBase.cuhk.edu.cn/) [54,55] the complete set of validated microRNA-target interactions (MTIs), irrespective of validation method used. miRTarBase is a literature-built database that contains MTIs experimentally-validated through different laboratory techniques, ranging from immunoblotting to sequencing-based approaches, and their related bibliography references. Then, the list extracted from miRTarBase was manually screened to select only gene reporter assay-based direct MTIs. We focused on luciferase reporter assay validation method as it represents the best approach to assess the univocal regulatory effects of a specific miRNA on its potential targets [56]. To date, no solid laboratory-based evidence of regulation has been described among miRs and JAG2, DLL3 and DLL4 mRNAs. ND, not determined; PMID, PubMed identifier.

**Table S2. Drugs and compounds targeting Notch pathway**

| **Drug**  **class** | **Drug**  **name** | **Drug description**  **and function** | **References** |
| --- | --- | --- | --- |
| **Notch**  **trafficking**  **inhibitors** | **FLI-06** | - β-annulated dihydropyridine;  - disturbs the intracellular trafficking of several secretory, GPI-anchored and transmembrane proteins, including the Notch receptors;  - first compound of a novel class of molecules interfering with the Notch secretory pathway at an early phase;  - disrupts Golgi apparatus and inhibits ER export, blocking cargo recruitment to ER exit sites;  - inhibits Notch pathway activation in tongue cancer cell lines, and in turn reduces tumour cells proliferation and self-renewal. | [57-60] |
| **Notch**  **ligand**  **antibodies** | **demcizumab**  **(OMP-21M18), enoticumab**  **(REGN421), MEDI0639** | - anti-DLL4 mAbs;  - used in cancer preclinical models and under clinical development (Phase I-II CTs) for the treatment of advanced solid cancers, often in combination with standard chemotherapy. CTs revealed that demcizumab is well-tolerated but lacks benefit over standard-of-care. | [61,62] |
|  | **Anti-JAG1 antibodies (*e.g.* J1-65D, J1-183D,**  **J1-156A)** | - anti-JAG1 mAbs targeting the receptor-binding site within the DSL domain of JAG1;  - growth-inhibitory effects in MDA-MB-231 breast cancer cell line xenografts. | [63] |
| **Notch**  **receptor**  **antibodies** | **Brontictuzumab (OMP-52M51)** | - anti-NOTCH1 IgG2 humanized mAb;  - selective binding of the NOTCH1 NRR;  - used in cancer preclinical models (*e.g.* cell lines and PDX) and under clinical development (Phase I CTs) for the treatment of haematological malignancies and solid tumours hallmarked by Notch activation. | [62,64,65] |
|  | **Tarextumab**  **(OMP-59R5)** | - mAb targeting NOTCH2 and NOTCH3;  - used in cancer preclinical models and under clinical development (Phase I-II CTs) for the treatment of haematological malignancies and solid tumours hallmarked by Notch activation.  - However, two CTs NCT01859741 (Phase Ib-II) and NCT01647828 (Phase II) for the treatment of NSCLC and metastatic pancreatic cancer failed to reach study endpoints (OR, OSS, PFS). | [62,66] |
| **Notch**  **receptor**  **and ligand**  **decoys** | **Notch decoys**  **(*e.g.* N1_1-13_, N1_10-24_, N1_1-24_)** | - soluble molecules containing from 10 to 36 extracellular EGF-like repeats of human NOTCH1 fused to human IgGγ heavy chain (Fc);  - Notch decoys interfere with the physiological Notch receptor-Notch ligand binding;  - the number and type of EGF repeats determine the decoy-specific inhibition of DLL- or JAG-mediated Notch activation;  - NOTCH1 decoys show anti-angiogenic effects in mouse cancer models. | [67,68] |
|  | **Dll4-Fc** | - soluble form of the DLL4 ligand fused to the IgG1 constant region (Dll4-Fc);  - blocks DLL4-mediated Notch activation;  - anti-tumorigenic effects (*e.g.* reduction of liver metastasis) in *in vitro* and *in vivo* models of SCLC. | [69] |
| **Small molecules uncoupling Notch-Jagged interactions** | **BAS 00693376 (IGOR1)**  **BAS 00327971**  **(IGOR2)** | - uncouplers of the NOTCH2:JAGGED2 interaction;  - IGOR1 inhibits Notch activation in multiple myeloma OPM-2 cells and significantly reduces their resistance to lenalidomide and melphalan – two standard drugs against MM. | [70,71] |
| **ADAM17**  **inhibitors** | **ZLDI-8** | - reduces ADAM17 activity and inhibits Notch processing/activation at the level of S2 cleavage;  - ZLDI-8 alleviates pro-tumorigenic processes (*e.g.* EMT, migration, proliferation) and reduces the expression of pro-survival genes in several preclinical cancer models (*e.g.* CRC, HCC, NSCLC cell lines). | [72-74] |
|  | **MEDI3622** | - anti-ADAM17 mAb;  - suppresses the growth of CRC PDXs. | [75,76] |
| **Gamma**  **Secretase**  **Inhibitors**  **(GSIs)** | **AL101**  **(BMS-906024)**  **AL102**  **(BMS-986115)** | - used in cancer preclinical models and under clinical development (Phase I-II CTs) for the treatment of haematological malignancies and advanced solid cancers, often in combination with standard chemotherapy. | [62,77] |
|  | **Crenigacestat**  **(LY3039478), LY411575** | - Crenigacestat is a γ-secretase inhibitor currently under clinical development (Phase I studies) for the treatment of a number of tumours, both as monotherapy and in combination with targeted agent or standard chemotherapy. | [78,79] |
|  | **DAPT** | - used in preclinical models of skin fibrosis, liquid malignancies and solid tumours (growth hormone-producing adenomas and osteosarcoma). | [80-83] |
|  | **Nirogacestat**  **(PF-03084014)** | - orally-administered drug;  - anti-tumour and anti-metastatic activity in preclinical studies and CTs;  - first Notch GSI entering a Phase III CT (*i.e.* NCT03785964 for the treatment of aggressive desmoid tumours/aggressive fibromatosis). | [62,84,85] |
|  | **RO4929097**  **(RG4733)** | - LY411575-derived compound;  - orally bioavailable;  - widely used in cancer preclinical models and in phase I-II CTs for the treatment of number of solid malignancies both as single agent as well as in combination with standard chemotherapy, radiotherapy and other molecular-targeted therapies;  - CTs demonstrate that RO4929097 has good tolerability but poor efficacy, leading to CT withdrawal. | [62,86] |
| **Inhibitors of the trimeric transcriptional complex** | **CB-103** | - used in preclinical models of cancer (*e.g.* T-ALL cell lines and PDXs) and under clinical investigation (phase I-IIA CT) for the treatment of solid tumours and blood malignancies (NCT03422679 and NCT04714619). | [87] |
|  | **IMR-1** | - disrupts MAML1 recruitment to the Notch transcriptional activation complex on the chromatin;  - reduces colony forming efficiency in different cancer cell lines and tumour growth in PDX models of oesophageal adenocarcinoma. | [88] |
|  | **RIN1**  **(RBPJ Inhibitor-1)** | - in the context of activated Notch signalling, inhibits the NOTCH2/3 ICD-mediated transcription of Hes1;  - disrupts the function of pre-synthesized RBPJ;  - RIN1 inhibits NOTCH-dependent tumour cell proliferation in cell lines bearing activating Notch mutations; | [89] |
|  | **SAHM1** | - peptide of SAHM family (SAHMs);  - SAHMs compete with MAML1 for the N1ICD–RBPJ complex formation and repress Notch-dependent gene expression *in vitro*;  - SAHM1 represses the NOTCH signalling program in human and murine T-ALL cells;  - SAHM1 inhibits allergic airway inflammation in an asthma mouse model;  - SAHM1 blocks Notch signalling and reduces proliferation rate in corneal epithelial stem cells (LSCs), without affecting cell stratification and differentiation. | [90-92] |
| **Repurposing drugs** | **Artesunate (ART)** | - member of a family of compounds derived from artemisin, a sesquiterpene endoperoxide obtained from *Artemisia annua L*, and used as anti-malarial drug;  - ART exerts antifibrotic effect in different preclinical models of fibrosis, including IPF;  - in IPF, ART inhibits Notch signalling pathway and the TGF-β1-mediated induction of α-SMA and type IV collagen both in *in vitro* and *in vivo*. | [93,94] |
|  | **Astragaloside (AS)** | - AS is the main component of the dried roots of *Astragalus membranaceus (Fisch.) Bunge* - one of the most widely used traditional Chinese herbal medicines;  - AS has been reported to alleviate hepatic and pulmonary fibrosis *in vivo*;  - in rats with BDL-induced liver fibrosis, AS oral administration reduces mRNA and protein levels of NOTCH2-4 and JAG1, whilst increases the expression levels of NUMB – a Notch inhibitor.  - in rats with BML-induced lung fibrosis, AS intraperitoneal injection reduces α-SMA, TGF-β1, JAG1 and NOTCH1 mRNA and protein levels. | [95,96] |
|  | **Scutellarin (SCU)** | - plant-derived flavonoid with antioxidant properties;  - SCU activates Notch signalling and reduces α-SMA levels in a rat model of myocardial fibrosis;  - SCU modulates Notch pathway members in astrocytes and microglia in a cell- and context-dependent manner. | [97-99] |
| **miRNA-based approaches** | **N1-34a-NPs** | - nanoparticles (NPs) functionalized with NOTCH1 antibodies (N1) containing miR-34a mimics (34a);  - used for the specific targeting of triple breast negative cancer (TBNC) cells hallmarked by NOTCH1 receptor over-expression and reduced levels of miR-34a;  - N1-34a-NPs reduce the expression levels of pro-tumorigenic miR-34a targets and Notch downstream genes (*e.g.* Hes5) in MDA-MB-231 TBNC cells;  - N1-34a-NP-treated TBNC cells show senescence and reduced cell proliferation and migration. | [100] |

**Abbreviations:** ADAM17, ADAM metallopeptidase domain 17; α-SMA, α-smooth muscle actin; BDL, bile duct ligation; BML, bleomycin; CRC, Colorectal cancer; CT, Clinical trial; DLL4, Delta like canonical Notch ligand 4; DSL, Delta/ Serrate/Lag2; EGF, epithelial growth factor; EMT, epithelial-mesenchymal transition; ER, endoplasmic reticulum;; GPI, Glycosylphosphatidylinositol; GSI, Gamma-secretase inhibitor; HCC, hepatocellular carcinoma; IPF, idiopathic pulmonary fibrosis; mAb, monoclonal antibody; MAML1, mastermind like transcriptional coactivator 1; MM, multiple myeloma; N1ICD, Notch 1 intracellular domain (similar abbreviations are used for Notch 2, Notch 3 and Notch 4 intracellular domains); NRR, negative regulatory region; NSCLC, non-small cell lung cancer; ORR, overall response rate; OS, overall survival; PFS, progression-free survival; SAHM, stapled α-helical peptides derived from MAML1; PDX, patient-derived xenograft; RBPJ, recombination signal binding protein for immunoglobulin kappa J region; SCLC, small cell lung cancer; T-ALL, T-cell acute lymphoblastic leukemia; TGF-β1, transforming growth factor-β1.

**References**

1. Qian L, Zhang W, Lei B, He A, Ye L, Li X, Dong X. MicroRNA-101 regulates T-cell acute lymphoblastic leukemia progression and chemotherapeutic sensitivity by targeting Notch1. Oncol Rep. 2016; 36(5):2511-16. 10.3892/or.2016.5117

2. Lin J, Teo S, Lam DH, Jeyaseelan K, Wang S. MicroRNA-10b pleiotropically regulates invasion, angiogenicity and apoptosis of tumor cells resembling mesenchymal subtype of glioblastoma multiforme. Cell Death Dis. 2012; 3:e398. 10.1038/cddis.2012.134

3. Zhang L, Dong Y, Zhu N, Tsoi H, Zhao Z, Wu CW, Wang K, Zheng S, Ng SS, Chan FK, Sung JJ, Yu J. microRNA-139-5p exerts tumor suppressor function by targeting NOTCH1 in colorectal cancer. Mol Cancer. 2014; 13:124. 10.1186/1476-4598-13-124

4. Zhang HD, Sun DW, Mao L, Zhang J, Jiang LH, Li J, Wu Y, Ji H, Chen W, Wang J, Ma R, Cao HX, Wu JZ, Tang JH. MiR-139-5p inhibits the biological function of breast cancer cells by targeting Notch1 and mediates chemosensitivity to docetaxel. Biochem Biophys Res Commun. 2015; 465(4):702-13. 10.1016/j.bbrc.2015.08.053

5. Xu K, Shen K, Liang X, Li Y, Nagao N, Li J, Liu J, Yin P. MiR-139-5p reverses CD44+/CD133+-associated multidrug resistance by downregulating NOTCH1 in colorectal carcinoma cells. Oncotarget. 2016; 7(46):75118-29. 10.18632/oncotarget.12611

6. Liu R, Li W, Wu C. A functional polymorphism in the premiR146a gene influences the prognosis of glioblastoma multiforme by interfering with the balance between Notch1 and Notch2. Mol Med Rep. 2015; 12(4):5475-81. 10.3892/mmr.2015.4067

7. Cama A, Verginelli F, Lotti LV, Napolitano F, Morgano A, D'Orazio A, Vacca M, Perconti S, Pepe F, Romani F, Vitullo F, di Lella F, Visone R, Mannelli M, Neumann HP, Raiconi G, Paties C, Moschetta A, Tagliaferri R, Veronese A, Sanna M, Mariani-Costantini R. Integrative genetic, epigenetic and pathological analysis of paraganglioma reveals complex dysregulation of NOTCH signaling. Acta Neuropathol. 2013; 126(4):575-94. 10.1007/s00401-013-1165-y

8. Ortega M, Bhatnagar H, Lin AP, Wang L, Aster JC, Sill H, Aguiar RC. A microRNA-mediated regulatory loop modulates NOTCH and MYC oncogenic signals in B- and T-cell malignancies. Leukemia. 2015; 29(4):968-76. 10.1038/leu.2014.302

9. Wu J, Zheng C, Fan Y, Zeng C, Chen Z, Qin W, Zhang C, Zhang W, Wang X, Zhu X, Zhang M, Zen K, Liu Z. Downregulation of microRNA-30 facilitates podocyte injury and is prevented by glucocorticoids. J Am Soc Nephrol. 2014; 25(1):92-104. 10.1681/ASN.2012111101

10. Kefas B, Comeau L, Floyd DH, Seleverstov O, Godlewski J, Schmittgen T, Jiang J, diPierro CG, Li Y, Chiocca EA, Lee J, Fine H, Abounader R, Lawler S, Purow B. The neuronal microRNA miR-326 acts in a feedback loop with notch and has therapeutic potential against brain tumors. J Neurosci. 2009; 29(48):15161-8. 10.1523/JNEUROSCI.4966-09.2009

11. Lewis BP, Shih IH, Jones-Rhoades MW, Bartel DP, Burge CB. Prediction of mammalian microRNA targets. Cell. 2003; 115(7):787-98. 10.1016/s0092-8674(03)01018-3

12. Li Y, Guessous F, Zhang Y, Dipierro C, Kefas B, Johnson E, Marcinkiewicz L, Jiang J, Yang Y, Schmittgen TD, Lopes B, Schiff D, Purow B, Abounader R. MicroRNA-34a inhibits glioblastoma growth by targeting multiple oncogenes. Cancer Res. 2009; 69(19):7569-76. 10.1158/0008-5472.CAN-09-0529

13. Pang RT, Leung CO, Ye TM, Liu W, Chiu PC, Lam KK, Lee KF, Yeung WS. MicroRNA-34a suppresses invasion through downregulation of Notch1 and Jagged1 in cervical carcinoma and choriocarcinoma cells. Carcinogenesis. 2010; 31(6):1037-44. 10.1093/carcin/bgq066

14. Du R, Sun W, Xia L, Zhao A, Yu Y, Zhao L, Wang H, Huang C, Sun S. Hypoxia-induced down-regulation of microRNA-34a promotes EMT by targeting the Notch signaling pathway in tubular epithelial cells. PLoS One. 2012; 7(2):e30771. 10.1371/journal.pone.0030771

15. Li XJ, Ji MH, Zhong SL, Zha QB, Xu JJ, Zhao JH, Tang JH. MicroRNA-34a modulates chemosensitivity of breast cancer cells to adriamycin by targeting Notch1. Arch Med Res. 2012; 43(7):514-21. 10.1016/j.arcmed.2012.09.007

16. Zhang C, Yao Z, Zhu M, Ma X, Shi T, Li H, Wang B, Ouyang J, Zhang X. Inhibitory effects of microRNA-34a on cell migration and invasion of invasive urothelial bladder carcinoma by targeting Notch1. J Huazhong Univ Sci Technolog Med Sci. 2012; 32(3):375-82. 10.1007/s11596-012-0065-z

17. Chen Q, Yang F, Guo M, Wen G, Zhang C, Luong le A, Zhu J, Xiao Q, Zhang L. miRNA-34a reduces neointima formation through inhibiting smooth muscle cell proliferation and migration. J Mol Cell Cardiol. 2015; 89(Pt A):75-86. 10.1016/j.yjmcc.2015.10.017

18. Tang Y, Tang Y, Cheng YS. miR-34a inhibits pancreatic cancer progression through Snail1-mediated epithelial-mesenchymal transition and the Notch signaling pathway. Sci Rep. 2017; 7:38232. 10.1038/srep38232

19. Wang XP, Zhou J, Han M, Chen CB, Zheng YT, He XS, Yuan XP. MicroRNA-34a regulates liver regeneration and the development of liver cancer in rats by targeting Notch signaling pathway. Oncotarget. 2017; 8(8):13264-76. 10.18632/oncotarget.14807

20. Bae Y, Yang T, Zeng HC, Campeau PM, Chen Y, Bertin T, Dawson BC, Munivez E, Tao J, Lee BH. miRNA-34c regulates Notch signaling during bone development. Hum Mol Genet. 2012; 21(13):2991-3000. 10.1093/hmg/dds129

21. Song B, Yan J, Liu C, Zhou H, Zheng Y. Tumor Suppressor Role of miR-363-3p in Gastric Cancer. Med Sci Monit. 2015; 21:4074-80. 10.12659/msm.896556

22. De Weer A, Van der Meulen J, Rondou P, Taghon T, Konrad TA, De Preter K, Mestdagh P, Van Maerken T, Van Roy N, Jeison M, Yaniv I, Cauwelier B, Noens L, Poirel HA, Vandenberghe P, Lambert F, De Paepe A, Sanchez MG, Odero M, Verhasselt B, Philippe J, Vandesompele J, Wieser R, Dastugue N, Van Vlierberghe P, Poppe B, Speleman F. EVI1-mediated down regulation of MIR449A is essential for the survival of EVI1 positive leukaemic cells. Br J Haematol. 2011; 154(3):337-48. 10.1111/j.1365-2141.2011.08737.x

23. Yan C, Yu J, Kang W, Liu Y, Ma Z, Zhou L. miR-935 suppresses gastric signet ring cell carcinoma tumorigenesis by targeting Notch1 expression. Biochem Biophys Res Commun. 2016; 470(1):68-74. 10.1016/j.bbrc.2015.12.116

24. Mohammadi-Yeganeh S, Mansouri A, Paryan M. Targeting of miR9/NOTCH1 interaction reduces metastatic behavior in triple-negative breast cancer. Chem Biol Drug Des. 2015; 86(5):1185-91. 10.1111/cbdd.12584

25. Chen L, Chen XR, Zhang R, Li P, Liu Y, Yan K, Jiang XD. MicroRNA-107 inhibits glioma cell migration and invasion by modulating Notch2 expression. J Neurooncol. 2013; 112(1):59-66. 10.1007/s11060-012-1037-7

26. Wang C, Zhang W, Zhang L, Chen X, Liu F, Zhang J, Guan S, Sun Y, Chen P, Wang D, Un Nesa E, Cheng Y, Yousef GM. miR-146a-5p mediates epithelial-mesenchymal transition of oesophageal squamous cell carcinoma via targeting Notch2. Br J Cancer. 2016; 115(12):1548-54. 10.1038/bjc.2016.367

27. Wang N, Chen FE, Long ZW. Mechanism of MicroRNA-146a/Notch2 Signaling Regulating IL-6 in Graves Ophthalmopathy. Cell Physiol Biochem. 2017; 41(4):1285-97. 10.1159/000464430

28. Polioudakis D, Abell NS, Iyer VR. MiR-191 Regulates Primary Human Fibroblast Proliferation and Directly Targets Multiple Oncogenes. PLoS One. 2015; 10(5):e0126535. 10.1371/journal.pone.0126535

29. Huang TT, Ping YH, Wang AM, Ke CC, Fang WL, Huang KH, Lee HC, Chi CW, Yeh TS. The reciprocal regulation loop of Notch2 pathway and miR-23b in controlling gastric carcinogenesis. Oncotarget. 2015; 6(20):18012-26. 10.18632/oncotarget.4000

30. Roese-Koerner B, Stappert L, Berger T, Braun NC, Veltel M, Jungverdorben J, Evert BO, Peitz M, Borghese L, Brustle O. Reciprocal Regulation between Bifunctional miR-9/9( *) and its Transcriptional Modulator Notch in Human Neural Stem Cell Self-Renewal and Differentiation. Stem Cell Reports. 2016; 7(2):207-19. 10.1016/j.stemcr.2016.06.008

31. Jeong JY, Kang H, Kim TH, Kim G, Heo JH, Kwon AY, Kim S, Jung SG, An HJ. MicroRNA-136 inhibits cancer stem cell activity and enhances the anti-tumor effect of paclitaxel against chemoresistant ovarian cancer cells by targeting Notch3. Cancer Lett. 2017; 386:168-78. 10.1016/j.canlet.2016.11.017

32. Gagan J, Dey BK, Layer R, Yan Z, Dutta A. Notch3 and Mef2c proteins are mutually antagonistic via Mkp1 protein and miR-1/206 microRNAs in differentiating myoblasts. J Biol Chem. 2012; 287(48):40360-70. 10.1074/jbc.M112.378414

33. Furukawa S, Kawasaki Y, Miyamoto M, Hiyoshi M, Kitayama J, Akiyama T. The miR-1-NOTCH3-Asef pathway is important for colorectal tumor cell migration. PLoS One. 2013; 8(11):e80609. 10.1371/journal.pone.0080609

34. Ghisi M, Corradin A, Basso K, Frasson C, Serafin V, Mukherjee S, Mussolin L, Ruggero K, Bonanno L, Guffanti A, De Bellis G, Gerosa G, Stellin G, D'Agostino DM, Basso G, Bronte V, Indraccolo S, Amadori A, Zanovello P. Modulation of microRNA expression in human T-cell development: targeting of NOTCH3 by miR-150. Blood. 2011; 117(26):7053-62. 10.1182/blood-2010-12-326629

35. Xu B, Zhang YW, Tong XH, Liu YS. Characterization of microRNA profile in human cumulus granulosa cells: Identification of microRNAs that regulate Notch signaling and are associated with PCOS. Mol Cell Endocrinol. 2015; 404:26-36. 10.1016/j.mce.2015.01.030

36. Zhang Q, Li Q, Xu T, Jiang H, Xu LG. miR-491-5p suppresses cell growth and invasion by targeting Notch3 in nasopharyngeal carcinoma. Oncol Rep. 2016; 35(6):3541-7. 10.3892/or.2016.4713

37. Hashimoto Y, Akiyama Y, Otsubo T, Shimada S, Yuasa Y. Involvement of epigenetically silenced microRNA-181c in gastric carcinogenesis. Carcinogenesis. 2010; 31(5):777-84. 10.1093/carcin/bgq013

38. Yu F, Jiao Y, Zhu Y, Wang Y, Zhu J, Cui X, Liu Y, He Y, Park EY, Zhang H, Lv X, Ma K, Su F, Park JH, Song E. MicroRNA 34c gene down-regulation via DNA methylation promotes self-renewal and epithelial-mesenchymal transition in breast tumor-initiating cells. J Biol Chem. 2012; 287(1):465-73. 10.1074/jbc.M111.280768

39. Ahluwalia JK, Soni K, Sivasubbu S, Brahmachari V. Modeling SNP mediated differential targeting of homologous 3'UTR by microRNA. RNA Biol. 2012; 9(3):351-60. 10.4161/rna.19318

40. Jiang L, Lin T, Xu C, Hu S, Pan Y, Jin R. miR-124 interacts with the Notch1 signalling pathway and has therapeutic potential against gastric cancer. J Cell Mol Med. 2016; 20(2):313-22. 10.1111/jcmm.12724

41. Liu MX, Siu MK, Liu SS, Yam JW, Ngan HY, Chan DW. Epigenetic silencing of microRNA-199b-5p is associated with acquired chemoresistance via activation of JAG1-Notch1 signaling in ovarian cancer. Oncotarget. 2014; 5(4):944-58. 10.18632/oncotarget.1458

42. Qu X, Chen Z, Fan D, Sun C, Zeng Y, Guo Z, Qi Q, Li W. MiR-199b-5p inhibits osteogenic differentiation in ligamentum flavum cells by targeting JAG1 and modulating the Notch signalling pathway. J Cell Mol Med. 2017; 21(6):1159-70. 10.1111/jcmm.13047

43. Hashimi ST, Fulcher JA, Chang MH, Gov L, Wang S, Lee B. MicroRNA profiling identifies miR-34a and miR-21 and their target genes JAG1 and WNT1 in the coordinate regulation of dendritic cell differentiation. Blood. 2009; 114(2):404-14. 10.1182/blood-2008-09-179150

44. Selcuklu SD, Donoghue MT, Kerin MJ, Spillane C. Regulatory interplay between miR-21, JAG1 and 17beta-estradiol (E2) in breast cancer cells. Biochem Biophys Res Commun. 2012; 423(2):234-9. 10.1016/j.bbrc.2012.05.074

45. Lu J, Song G, Tang Q, Yin J, Zou C, Zhao Z, Xie X, Xu H, Huang G, Wang J, Lee DF, Khokha R, Yang H, Shen J. MiR-26a inhibits stem cell-like phenotype and tumor growth of osteosarcoma by targeting Jagged1. Oncogene. 2017; 36(2):231-41. 10.1038/onc.2016.194

46. Shi L, Yin W, Zhang Z, Shi G. Down-regulation of miR-26b induces cisplatin resistance in nasopharyngeal carcinoma by repressing JAG1. FEBS Open Bio. 2016; 6(12):1211-19. 10.1002/2211-5463.12135

47. Chen L, Holmstrom K, Qiu W, Ditzel N, Shi K, Hokland L, Kassem M. MicroRNA-34a inhibits osteoblast differentiation and in vivo bone formation of human stromal stem cells. Stem Cells. 2014; 32(4):902-12. 10.1002/stem.1615

48. Lee YM, Lee JY, Ho CC, Hong QS, Yu SL, Tzeng CR, Yang PC, Chen HW. miRNA-34b as a tumor suppressor in estrogen-dependent growth of breast cancer cells. Breast Cancer Res. 2011; 13(6):R116. 10.1186/bcr3059

49. Li J, Dong S, Ye M, Peng G, Luo J, Wang C, Wang J, Zhao Q, Chang Y, Wang H. MicroRNA-489-3p Represses Hepatic Stellate Cells Activation by Negatively Regulating the JAG1/Notch3 Signaling Pathway. Dig Dis Sci. 2021; 66(1):143-50. 10.1007/s10620-020-06174-w

50. Chen L, Zhang W, Yan W, Han L, Zhang K, Shi Z, Zhang J, Wang Y, Li Y, Yu S, Pu P, Jiang C, Jiang T, Kang C. The putative tumor suppressor miR-524-5p directly targets Jagged-1 and Hes-1 in glioma. Carcinogenesis. 2012; 33(11):2276-82. 10.1093/carcin/bgs261

51. Shui Y, Yu X, Duan R, Bao Q, Wu J, Yuan H, Ma C. miR-130b-3p inhibits cell invasion and migration by targeting the Notch ligand Delta-like 1 in breast carcinoma. Gene. 2017; 609:80-87. 10.1016/j.gene.2017.01.036

52. Takane K, Fujishima K, Watanabe Y, Sato A, Saito N, Tomita M, Kanai A. Computational prediction and experimental validation of evolutionarily conserved microRNA target genes in bilaterian animals. BMC Genomics. 2010; 11:101. 10.1186/1471-2164-11-101

53. Pang RT, Leung CO, Lee CL, Lam KK, Ye TM, Chiu PC, Yeung WS. MicroRNA-34a is a tumor suppressor in choriocarcinoma via regulation of Delta-like1. BMC Cancer. 2013; 13:25. 10.1186/1471-2407-13-25

54. Hsu SD, Lin FM, Wu WY, Liang C, Huang WC, Chan WL, Tsai WT, Chen GZ, Lee CJ, Chiu CM, Chien CH, Wu MC, Huang CY, Tsou AP, Huang HD. miRTarBase: a database curates experimentally validated microRNA-target interactions. Nucleic Acids Res. 2011; 39(Database issue):D163-9. 10.1093/nar/gkq1107

55. Huang HY, Lin YC, Li J, Huang KY, Shrestha S, Hong HC, Tang Y, Chen YG, Jin CN, Yu Y, Xu JT, Li YM, Cai XX, Zhou ZY, Chen XH, Pei YY, Hu L, Su JJ, Cui SD, Wang F, Xie YY, Ding SY, Luo MF, Chou CH, Chang NW, Chen KW, Cheng YH, Wan XH, Hsu WL, Lee TY, Wei FX, Huang HD. miRTarBase 2020: updates to the experimentally validated microRNA-target interaction database. Nucleic Acids Res. 2020; 48(D1):D148-D54. 10.1093/nar/gkz896

56. Tomasello L, Cluts L, Croce CM. Experimental Validation of MicroRNA Targets: Luciferase Reporter Assay. Methods Mol Biol. 2019; 1970:315-30. 10.1007/978-1-4939-9207-2_17

57. Kramer A, Mentrup T, Kleizen B, Rivera-Milla E, Reichenbach D, Enzensperger C, Nohl R, Tauscher E, Gorls H, Ploubidou A, Englert C, Werz O, Arndt HD, Kaether C. Small molecules intercept Notch signaling and the early secretory pathway. Nat Chem Biol. 2013; 9(11):731-8. 10.1038/nchembio.1356

58. Yonemura Y, Li X, Muller K, Kramer A, Atigbire P, Mentrup T, Feuerhake T, Kroll T, Shomron O, Nohl R, Arndt HD, Hoischen C, Hemmerich P, Hirschberg K, Kaether C. Inhibition of cargo export at ER exit sites and the trans-Golgi network by the secretion inhibitor FLI-06. J Cell Sci. 2016; 129(20):3868-77. 10.1242/jcs.186163

59. Gomez-Galeno JE, Hurtado C, Cheng J, Yardimci C, Mercola M, Cashman JR. b-Annulated 1,4-dihydropyridines as Notch inhibitors. Bioorg Med Chem Lett. 2018; 28(20):3363-67. 10.1016/j.bmcl.2018.09.002

60. Gan RH, Lin LS, Xie J, Huang L, Ding LC, Su BH, Peng XE, Zheng DL, Lu YG. FLI-06 Intercepts Notch Signaling And Suppresses The Proliferation And Self-renewal Of Tongue Cancer Cells. Onco Targets Ther. 2019; 12:7663-74. 10.2147/OTT.S221231

61. Jenkins DW, Ross S, Veldman-Jones M, Foltz IN, Clavette BC, Manchulenko K, Eberlein C, Kendrew J, Petteruti P, Cho S, Damschroder M, Peng L, Baker D, Smith NR, Weir HM, Blakey DC, Bedian V, Barry ST. MEDI0639: a novel therapeutic antibody targeting Dll4 modulates endothelial cell function and angiogenesis in vivo. Mol Cancer Ther. 2012; 11(8):1650-60. 10.1158/1535-7163.MCT-11-1027

62. Moore G, Annett S, McClements L, Robson T. Top Notch Targeting Strategies in Cancer: A Detailed Overview of Recent Insights and Current Perspectives. Cells. 2020; 9(6). 10.3390/cells9061503

63. Masiero M, Li D, Whiteman P, Bentley C, Greig J, Hassanali T, Watts S, Stribbling S, Yates J, Bealing E, Li JL, Chillakuri C, Sheppard D, Serres S, Sarmiento-Soto M, Larkin J, Sibson NR, Handford PA, Harris AL, Banham AH. Development of Therapeutic Anti-JAGGED1 Antibodies for Cancer Therapy. Mol Cancer Ther. 2019; 18(11):2030-42. 10.1158/1535-7163.MCT-18-1176

64. Agnusdei V, Minuzzo S, Frasson C, Grassi A, Axelrod F, Satyal S, Gurney A, Hoey T, Seganfreddo E, Basso G, Valtorta S, Moresco RM, Amadori A, Indraccolo S. Therapeutic antibody targeting of Notch1 in T-acute lymphoblastic leukemia xenografts. Leukemia. 2014; 28(2):278-88. 10.1038/leu.2013.183

65. Ferrarotto R, Eckhardt G, Patnaik A, LoRusso P, Faoro L, Heymach JV, Kapoun AM, Xu L, Munster P. A phase I dose-escalation and dose-expansion study of brontictuzumab in subjects with selected solid tumors. Ann Oncol. 2018; 29(7):1561-68. 10.1093/annonc/mdy171

66. Yen WC, Fischer MM, Axelrod F, Bond C, Cain J, Cancilla B, Henner WR, Meisner R, Sato A, Shah J, Tang T, Wallace B, Wang M, Zhang C, Kapoun AM, Lewicki J, Gurney A, Hoey T. Targeting Notch signaling with a Notch2/Notch3 antagonist (tarextumab) inhibits tumor growth and decreases tumor-initiating cell frequency. Clin Cancer Res. 2015; 21(9):2084-95. 10.1158/1078-0432.CCR-14-2808

67. Funahashi Y, Hernandez SL, Das I, Ahn A, Huang J, Vorontchikhina M, Sharma A, Kanamaru E, Borisenko V, Desilva DM, Suzuki A, Wang X, Shawber CJ, Kandel JJ, Yamashiro DJ, Kitajewski J. A notch1 ectodomain construct inhibits endothelial notch signaling, tumor growth, and angiogenesis. Cancer Res. 2008; 68(12):4727-35. 10.1158/0008-5472.CAN-07-6499

68. Kangsamaksin T, Murtomaki A, Kofler NM, Cuervo H, Chaudhri RA, Tattersall IW, Rosenstiel PE, Shawber CJ, Kitajewski J. NOTCH decoys that selectively block DLL/NOTCH or JAG/NOTCH disrupt angiogenesis by unique mechanisms to inhibit tumor growth. Cancer Discov. 2015; 5(2):182-97. 10.1158/2159-8290.CD-14-0650

69. Kuramoto T, Goto H, Mitsuhashi A, Tabata S, Ogawa H, Uehara H, Saijo A, Kakiuchi S, Maekawa Y, Yasutomo K, Hanibuchi M, Akiyama S, Sone S, Nishioka Y. Dll4-Fc, an inhibitor of Dll4-notch signaling, suppresses liver metastasis of small cell lung cancer cells through the downregulation of the NF-kappaB activity. Mol Cancer Ther. 2012; 11(12):2578-87. 10.1158/1535-7163.MCT-12-0640

70. Platonova N, Parravicini C, Sensi C, Paoli A, Colombo M, Neri A, Eberini I, Chiaramonte R. Identification of small molecules uncoupling the Notch::Jagged interaction through an integrated high-throughput screening. PLoS One. 2017; 12(11):e0182640. 10.1371/journal.pone.0182640

71. Colombo M, Garavelli S, Mazzola M, Platonova N, Giannandrea D, Colella R, Apicella L, Lancellotti M, Lesma E, Ancona S, Palano MT, Barbieri M, Taiana E, Lazzari E, Basile A, Turrini M, Pistocchi A, Neri A, Chiaramonte R. Multiple myeloma exploits Jagged1 and Jagged2 to promote intrinsic and bone marrow-dependent drug resistance. Haematologica. 2020; 105(7):1925-36. 10.3324/haematol.2019.221077

72. Li DD, Zhao CH, Ding HW, Wu Q, Ren TS, Wang J, Chen CQ, Zhao QC. A novel inhibitor of ADAM17 sensitizes colorectal cancer cells to 5-Fluorouracil by reversing Notch and epithelial-mesenchymal transition in vitro and in vivo. Cell Prolif. 2018; 51(5):e12480. 10.1111/cpr.12480

73. Zhang Y, Li D, Jiang Q, Cao S, Sun H, Chai Y, Li X, Ren T, Yang R, Feng F, Li BA, Zhao Q. Novel ADAM-17 inhibitor ZLDI-8 enhances the in vitro and in vivo chemotherapeutic effects of Sorafenib on hepatocellular carcinoma cells. Cell Death Dis. 2018; 9(7):743. 10.1038/s41419-018-0804-6

74. Lu HY, Zu YX, Jiang XW, Sun XT, Liu TY, Li RL, Wu Q, Zhang YS, Zhao QC. Novel ADAM-17 inhibitor ZLDI-8 inhibits the proliferation and metastasis of chemo-resistant non-small-cell lung cancer by reversing Notch and epithelial mesenchymal transition in vitro and in vivo. Pharmacol Res. 2019; 148:104406. 10.1016/j.phrs.2019.104406

75. Peng L, Cook K, Xu L, Cheng L, Damschroder M, Gao C, Wu H, Dall'Acqua WF. Molecular basis for the mechanism of action of an anti-TACE antibody. MAbs. 2016; 8(8):1598-605. 10.1080/19420862.2016.1226716

76. Dosch J, Ziemke E, Wan S, Luker K, Welling T, Hardiman K, Fearon E, Thomas S, Flynn M, Rios-Doria J, Hollingsworth R, Herbst R, Hurt E, Sebolt-Leopold J. Targeting ADAM17 inhibits human colorectal adenocarcinoma progression and tumor-initiating cell frequency. Oncotarget. 2017; 8(39):65090-99. 10.18632/oncotarget.17780

77. Gavai AV, Quesnelle C, Norris D, Han WC, Gill P, Shan W, Balog A, Chen K, Tebben A, Rampulla R, Wu DR, Zhang Y, Mathur A, White R, Rose A, Wang H, Yang Z, Ranasinghe A, D'Arienzo C, Guarino V, Xiao L, Su C, Everlof G, Arora V, Shen DR, Cvijic ME, Menard K, Wen ML, Meredith J, Trainor G, Lombardo LJ, Olson R, Baran PS, Hunt JT, Vite GD, Fischer BS, Westhouse RA, Lee FY. Discovery of Clinical Candidate BMS-906024: A Potent Pan-Notch Inhibitor for the Treatment of Leukemia and Solid Tumors. ACS Med Chem Lett. 2015; 6(5):523-7. 10.1021/acsmedchemlett.5b00001

78. Massard C, Azaro A, Soria JC, Lassen U, Le Tourneau C, Sarker D, Smith C, Ohnmacht U, Oakley G, Patel BKR, Yuen ESM, Benhadji KA, Rodon J. First-in-human study of LY3039478, an oral Notch signaling inhibitor in advanced or metastatic cancer. Ann Oncol. 2018; 29(9):1911-17. 10.1093/annonc/mdy244

79. Yuen E, Posada M, Smith C, Thorn K, Greenwood D, Burgess M, K AB, Ortega D, Chinchen L, Suico J. Evaluation of the effects of an oral notch inhibitor, crenigacestat (LY3039478), on QT interval, and bioavailability studies conducted in healthy subjects. Cancer Chemother Pharmacol. 2019; 83(3):483-92. 10.1007/s00280-018-3750-1

80. Dovey HF, John V, Anderson JP, Chen LZ, de Saint Andrieu P, Fang LY, Freedman SB, Folmer B, Goldbach E, Holsztynska EJ, Hu KL, Johnson-Wood KL, Kennedy SL, Kholodenko D, Knops JE, Latimer LH, Lee M, Liao Z, Lieberburg IM, Motter RN, Mutter LC, Nietz J, Quinn KP, Sacchi KL, Seubert PA, Shopp GM, Thorsett ED, Tung JS, Wu J, Yang S, Yin CT, Schenk DB, May PC, Altstiel LD, Bender MH, Boggs LN, Britton TC, Clemens JC, Czilli DL, Dieckman-McGinty DK, Droste JJ, Fuson KS, Gitter BD, Hyslop PA, Johnstone EM, Li WY, Little SP, Mabry TE, Miller FD, Audia JE. Functional gamma-secretase inhibitors reduce beta-amyloid peptide levels in brain. J Neurochem. 2001; 76(1):173-81. 10.1046/j.1471-4159.2001.00012.x

81. Geling A, Steiner H, Willem M, Bally-Cuif L, Haass C. A gamma-secretase inhibitor blocks Notch signaling in vivo and causes a severe neurogenic phenotype in zebrafish. EMBO Rep. 2002; 3(7):688-94. 10.1093/embo-reports/kvf124

82. Dai G, Deng S, Guo W, Yu L, Yang J, Zhou S, Gao T. Notch pathway inhibition using DAPT, a gamma-secretase inhibitor (GSI), enhances the antitumor effect of cisplatin in resistant osteosarcoma. Mol Carcinog. 2019; 58(1):3-18. 10.1002/mc.22873

83. Feng J, Wang J, Liu Q, Li J, Zhang Q, Zhuang Z, Yao X, Liu C, Li Y, Cao L, Li C, Gong L, Li D, Zhang Y, Gao H. DAPT, a gamma-Secretase Inhibitor, Suppresses Tumorigenesis, and Progression of Growth Hormone-Producing Adenomas by Targeting Notch Signaling. Front Oncol. 2019; 9:809. 10.3389/fonc.2019.00809

84. Kummar S, O'Sullivan Coyne G, Do KT, Turkbey B, Meltzer PS, Polley E, Choyke PL, Meehan R, Vilimas R, Horneffer Y, Juwara L, Lih A, Choudhary A, Mitchell SA, Helman LJ, Doroshow JH, Chen AP. Clinical Activity of the gamma-Secretase Inhibitor PF-03084014 in Adults With Desmoid Tumors (Aggressive Fibromatosis). J Clin Oncol. 2017; 35(14):1561-69. 10.1200/JCO.2016.71.1994

85. Takahashi T, Prensner JR, Robson CD, Janeway KA, Weigel BJ. Safety and efficacy of gamma-secretase inhibitor nirogacestat (PF-03084014) in desmoid tumor: Report of four pediatric/young adult cases. Pediatr Blood Cancer. 2020; 67(10):e28636. 10.1002/pbc.28636

86. Luistro L, He W, Smith M, Packman K, Vilenchik M, Carvajal D, Roberts J, Cai J, Berkofsky-Fessler W, Hilton H, Linn M, Flohr A, Jakob-Rotne R, Jacobsen H, Glenn K, Heimbrook D, Boylan JF. Preclinical profile of a potent gamma-secretase inhibitor targeting notch signaling with in vivo efficacy and pharmacodynamic properties. Cancer Res. 2009; 69(19):7672-80. 10.1158/0008-5472.CAN-09-1843

87. Lehal R, Zaric J, Vigolo M, Urech C, Frismantas V, Zangger N, Cao L, Berger A, Chicote I, Loubery S, Choi SH, Koch U, Blacklow SC, Palmer HG, Bornhauser B, Gonzalez-Gaitan M, Arsenijevic Y, Zoete V, Aster JC, Bourquin JP, Radtke F. Pharmacological disruption of the Notch transcription factor complex. Proc Natl Acad Sci U S A. 2020; 117(28):16292-301. 10.1073/pnas.1922606117

88. Astudillo L, Da Silva TG, Wang Z, Han X, Jin K, VanWye J, Zhu X, Weaver K, Oashi T, Lopes PE, Orton D, Neitzel LR, Lee E, Landgraf R, Robbins DJ, MacKerell AD, Jr., Capobianco AJ. The Small Molecule IMR-1 Inhibits the Notch Transcriptional Activation Complex to Suppress Tumorigenesis. Cancer Res. 2016; 76(12):3593-603. 10.1158/0008-5472.CAN-16-0061

89. Hurtado C, Safarova A, Smith M, Chung R, Bruyneel AAN, Gomez-Galeno J, Oswald F, Larson CJ, Cashman JR, Ruiz-Lozano P, Janiak P, Suzuki T, Mercola M. Disruption of NOTCH signaling by a small molecule inhibitor of the transcription factor RBPJ. Sci Rep. 2019; 9(1):10811. 10.1038/s41598-019-46948-5

90. Moellering RE, Cornejo M, Davis TN, Del Bianco C, Aster JC, Blacklow SC, Kung AL, Gilliland DG, Verdine GL, Bradner JE. Direct inhibition of the NOTCH transcription factor complex. Nature. 2009; 462(7270):182-8. 10.1038/nature08543

91. KleinJan A, Tindemans I, Montgomery JE, Lukkes M, de Bruijn MJW, van Nimwegen M, Bergen I, Moellering RE, Hoogsteden HC, Boon L, Amsen D, Hendriks RW. The Notch pathway inhibitor stapled alpha-helical peptide derived from mastermind-like 1 (SAHM1) abrogates the hallmarks of allergic asthma. J Allergy Clin Immunol. 2018; 142(1):76-85 e8. 10.1016/j.jaci.2017.08.042

92. Gonzalez S, Uhm H, Deng SX. Notch Inhibition Prevents Differentiation of Human Limbal Stem/Progenitor Cells in vitro. Sci Rep. 2019; 9(1):10373. 10.1038/s41598-019-46793-6

93. Liu Y, Huang G, Mo B, Wang C. Artesunate ameliorates lung fibrosis via inhibiting the Notch signaling pathway. Exp Ther Med. 2017; 14(1):561-66. 10.3892/etm.2017.4573

94. Wang Y, Wang Y, You F, Xue J. Novel use for old drugs: The emerging role of artemisinin and its derivatives in fibrosis. Pharmacol Res. 2020; 157:104829. 10.1016/j.phrs.2020.104829

95. Yongping M, Zhang X, Xuewei L, Fan W, Chen J, Zhang H, Chen G, Liu C, Liu P. Astragaloside prevents BDL-induced liver fibrosis through inhibition of notch signaling activation. J Ethnopharmacol. 2015; 169:200-9. 10.1016/j.jep.2015.04.015

96. Zhou Y, Liao S, Zhang Z, Wang B, Wan L. Astragalus injection attenuates bleomycin-induced pulmonary fibrosis via down-regulating Jagged1/Notch1 in lungs. J Pharm Pharmacol. 2016; 68(3):389-96. 10.1111/jphp.12518

97. Zhou H, Chen X, Chen L, Zhou X, Zheng G, Zhang H, Huang W, Cai J. Anti-fibrosis effect of scutellarin via inhibition of endothelial-mesenchymal transition on isoprenaline-induced myocardial fibrosis in rats. Molecules. 2014; 19(10):15611-23. 10.3390/molecules191015611

98. Fang M, Yuan Y, Rangarajan P, Lu J, Wu Y, Wang H, Wu C, Ling EA. Scutellarin regulates microglia-mediated TNC1 astrocytic reaction and astrogliosis in cerebral ischemia in the adult rats. BMC Neurosci. 2015; 16:84. 10.1186/s12868-015-0219-6

99. Yuan Y, Rangarajan P, Kan EM, Wu Y, Wu C, Ling EA. Scutellarin regulates the Notch pathway and affects the migration and morphological transformation of activated microglia in experimentally induced cerebral ischemia in rats and in activated BV-2 microglia. J Neuroinflammation. 2015; 12:11. 10.1186/s12974-014-0226-z

100. Valcourt DM, Day ES. Dual Regulation of miR-34a and Notch Signaling in Triple-Negative Breast Cancer by Antibody/miRNA Nanocarriers. Mol Ther Nucleic Acids. 2020; 21:290-98. 10.1016/j.omtn.2020.06.003
